# Genetic Affinities and Adaptation of the South-West Coast Populations of India

**DOI:** 10.1093/gbe/evad225

**Published:** 2023-12-11

**Authors:** Lomous Kumar, Anuhya Chowdhari, Jaison J Sequeira, Mohammed S Mustak, Moinak Banerjee, Kumarasamy Thangaraj

**Affiliations:** CSIR-Centre for Cellular and Molecular Biology, Hyderabad 500007, India; CSIR-Centre for Cellular and Molecular Biology, Hyderabad 500007, India; Department of Applied Zoology, Mangalore University, Mangalore 574199, India; Department of Applied Zoology, Mangalore University, Mangalore 574199, India; Human Molecular Genetics Laboratory, Rajiv Gandhi Centre for Biotechnology, Thiruvananthapuram 695014, Kerala, India; CSIR-Centre for Cellular and Molecular Biology, Hyderabad 500007, India

**Keywords:** South-west coast, Indo-Europeans, autosomal SNP, Godavari basin, feudal lord, Gangetic plain

## Abstract

Evolutionary event has not only altered the genetic structure of human populations but also associated with social and cultural transformation. South Asian populations were the result of migration and admixture of genetically and culturally diverse groups. Most of the genetic studies pointed to large-scale admixture events between Ancestral North Indian (ANI) and Ancestral South Indian (ASI) groups, also additional layers of recent admixture. In the present study, we have analyzed 213 individuals inhabited in South-west coast India with traditional warriors and feudal lord status and historically associated with migratory events from North/North West India and possible admixture with West Eurasian populations, whose genetic links are still missing. Analysis of autosomal Single Nucleotide Polymorphism (SNP) markers suggests that these groups possibly derived their ancestry from some groups of North West India having additional Middle Eastern genetic components. Higher distribution of West Eurasian mitochondrial haplogroups also points to female-mediated admixture. Estimation of Effective Migration Surface (EEMS) analysis indicates Central India and Godavari basin as a crucial transition zone for population migration from North and North West India to South-west coastal India. Selection screen using 3 distinct outlier-based approaches revealed genetic signatures related to Immunity and protection from Viral infections. Thus, our study suggests that the South-west coastal groups with traditional warriors and feudal lords’ status are of a distinct lineage compared to Dravidian and Gangetic plain Indo-Europeans and are remnants of very early migrations from North West India following the Godavari basin to Karnataka and Kerala.

**Significance**

Till date, genetic studies in South-west India have been done on groups that have migrated to India in the recent past, including; Siddis, Parsis, Jews, and Roman Catholics. Nevertheless, origin and affinities of many groups of South-west coast India, including populations with warrior or feudal lord status and historically mentioned as remnants of later migrations such as Indo-Scythians, Saka, or Kushans are unrevealed. Therefore, in this study, we have analyzed both mitochondrial and autosomal markers of the Warrior groups from South-west India and found that the South-west Indian populations represent an early lineage of non-Brahmin population with typical Ancestral North Indian-Ancestral South Indian (ANI-ASI) admixture along with additional Middle Eastern genetic component, unlike other Indo-Europeans or Dravidian caste groups. We also traced the possible migration route of South-west coast population, following the Godavari basin and signals of positive selection in the region.

## Introduction

South-west coast of India, which includes Konkan and Malabar region, is home to enormous cultural, linguistic, and religious diversity; emerging from over a millennium of migration, admixture, and cultural assimilation and development. This highly diverse region also harbors several caste groups linguistically belonging to either Dravidian family (Malayali and Tulu) or Konkani branch of Indo-European language family and historically falling under priestly (Havik and Hoysala), warrior (Nair and Thiyya) and landlord (Bunt) status. Historical records relate the origin of South-west coastal populations to ancient migration of people either from North West India or from the region near the Gangetic plain (Ahichhetra) (Fuller 1976). Ahichhatra is an Iron age archeological site of Painted Grayware (PGW) Culture in Gangetic plains of North India. According to an anthropologist (C. J. Fuller), both Nair and Bunts might have a common origin from Ahichhatra. Nair and Bunts, along with Nambudhiri and Tulu Brahmins were brought to the west coast very early during 375 CE by Kadamba King Mayura Varma (Fuller 1976). In the long history of the region different dynasties; including Kadamba, Chalukya, Rashtrakuta, and Alupa have used these groups as soldiers. There is a similar kind of mentioning in historical texts, such as *Keralolpathi* (Mangalore 2018) and Tulunadu *Grama Paddathi* (Saletore 1936), where they were mentioned as Naga warriors. Thiyya and Ezhava of Malabar also have a separate claims about their warrior status. Although historians believe that Thiyya and Ezhava have migrated from Sri Lanka, bringing palm cultivation and mainly involved in toddy tapping and agricultural works, some records (Pillai 1970) suggest that they were from Villavars of Chera dynasty. Bunts, Nairs, and some sects of Thiyya and Ezhava practice matriarchy even today. Historical evidence suggests that they have contact with populations from Middle East since Kadamba dynasty period (345 to 525 CE) and with Europeans later in history. Nature of these contacts was majorly commercial but had an impact on the society through the spread of religions like Judaism, Islam, and Christianity.

Previous genetic studies based on Y chromosomal microsatellite markers found more West Eurasian or North West Indian genetic influence in gene pool of Nairs and Ezhava (Nair et al. 2011; Mahal and Matsoukas 2018). While another genetic study based on the human leukocyte antigen (HLA)-A, -B, and -C diversity found greater Dravidian influence along with traces of admixture with West Eurasian, Mediterranean, Central Asian, and East Asian populations (Thomas et al. 2006). Previous genetic studies points to both West Eurasian as well as local Dravidian influence in the gene pool of South-west coast populations. However, earlier conclusions were based on low-resolution genetic markers. Therefore, no consensus exists among historians and geneticists of the South-west region regarding the actual origin of the populations of warrior class. Hence, we for the first time performed autosomal Single Nucleotide Polymorphism (SNP) genotyping and complete mitochondrial DNA sequence of South-west coast Indian groups to dissect the history of their genetic origin and adaptation, implementing various population genetic analysis approaches.

## Results

### Distinct Clustering of South-West Coastal Populations in the Context of Eurasian Populations

**Fig. 1.**
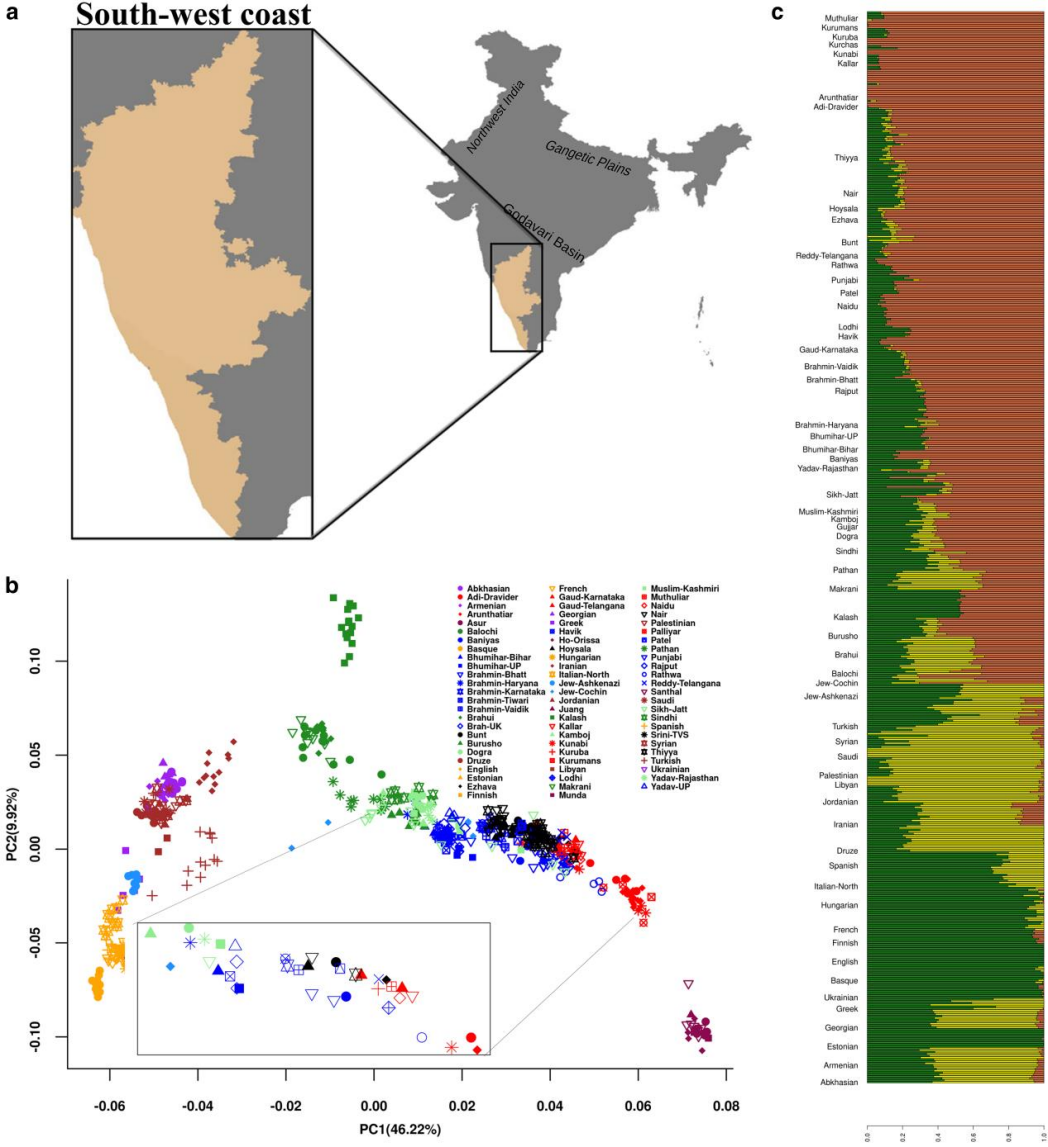
Sampled region, PCA and admixture plots. a) Map of India with regions of Konkan and Malabar coast from state of Karnataka and Kerala respectively (shaded with yellow color representing South-west coast), inset image shows South-west coast of Konkan and Malabar from Karnataka and Kerala. b) Biplot of PCA of South-west coastal groups with modern Eurasian populations with first 2 components. Inset is biplot of the population mean of first 2 principal components. c) Stacked bar plot of the ADMIXTURE analysis with *K* = 3, using modern west Eurasian populations as reference. Populations are arranged from bottom to top.

We first performed Principal Component Analysis (PCA) to gain insight into population structure. In PCA biplot, we found that the South Asian populations are distributed along the Ancestral North Indian-Ancestral South Indian (ANI-ASI) cline, with groups from Pakistan/North West India at one extreme (Balochi, Pathan, and Sindhi from Pakistan in Darkgreen/Yadav_Rajasthan, Sikh_Jatt, and Mushlim_Kashmiri from Northwest India in Lightgreen color); while many Dravidian tribes at the other extreme (Kurchas, Kurumans and in Red color) (see the legend of Fig. 1b). Most of the Gangetic plain Indo-Europeans (represented as Blue color) along with a few North West Indian individuals follow Pakistan/NWI (North West India) groups in the cline, while a heterogenous group follows these Gangetic plain Indo-Europeans in the cline, which includes Indo-European castes (nonpriestly status) from North India, groups with priestly status from Konkan, our 5 study groups (Nair, Thiyya, Bunt, Ezhava, and Hoysala; represented in Black color) and some of the Dravidian groups from geographical vicinity of our study groups. Of the study group, Nair and Hoysala clusters near Havik and Karnataka Brahmins, and much closer to the group of Gangetic plain Indo Europeans (see the legend of Fig. 1b). Bunt is adjacent to this group of Brahmins but further away in the cline. Thiyya individuals are found both along with Nairs and majority are further away toward Dravidian groups of this cluster along with Karnataka Gaud. Ezhava is last in this group with Reddy caste and some of the Dravidian populations, such as Kuruba and Kunabi. We also found an interesting displacement of a few Sikh_Jatt and Muslim_Kashmiri individuals toward this fourth heterogenous cluster of our study groups (Nair, Bunt, Thiyya, Ezhava, and Hoysala) (Fig. 1b).

In order to infer the ancestral genetic components in the context of modern Eurasians and to further enquire about the clustering pattern found in Principal Component (PC) analysis, we used model-based approach in ADMIXTURE (Alexander et al. 2009). Surprisingly, population groups placed in the heterogenous cluster in PC analysis, except Dravidian groups (Kuruba and Kunabi), showed additional prominence of yellow color in bar plot, which is characteristic of populations from Middle East and present in significant proportions among populations from Pakistan (Pathan, Balochi, and Makrani) and North West India (Kamboj, Gujjar, Muslim_Kashmiri, Dogra, and Yadav_Rajasthan) (Fig. 1c). Whereas, other populations in the ANI-ASI cline, such as Gangetic plain Indo-Europeans (Brahmin_Tiwari, Bhumihar_Bihar, Patel, and Lodhi) and Dravidian castes and tribe groups are lacking this component.

We further tested for the admixture history and allele sharing pattern in the 5 study groups of South-west coast by utilizing admixture F3 and Dstatistics methods in qp3Pop and qpDstat tools of ADMIXTOOLS 2 package in R. Admixture F3 was run in the form F3 (X, Palliyar; Nair/Bunt/Thiyya/Ezhava/Hoysala) using Palliyar as proxy for ASI source of ancestry and population X as different West Eurasians and South Asian populations. We found that Nair and Thiyya show significant F3 statistics with Middle Eastern population (Iranian, Druze) contrary to populations from either Caucasus or Europe, which is a characteristic of most of the groups with Indo-European affinity (supplementary fig. S1a and b, Supplementary Material online) (supplementary tables S2 and S3, Supplementary Material online). Bunt, Hoysala, and Ezhava populations show highest F3 statistics with either Caucasus or European populations, but groups from Middle East rank third or fourth in terms of higher F3 statistics (Iranian, Druze) (supplementary fig. S1c to e, Supplementary Material online) (supplementary tables S4 to S6, Supplementary Material online). However, none of the 5 populations (Nair, Bunt, Thiyya, Ezhava, and Hysala) that we have studied have shown significant admixture of F3 statistics with each other.

We then calculated Dstatistics in the form F4 (pop1, pop2, Steppe/Yamnaya, Yoruba) and F4 (pop1, pop2, Iran_N, Yoruba) to compare relative gene flow of various modern Indian populations from Steppe and Iranian-related ancestry in comparison to our study groups. Here, pop1 is various Indian cline groups and pop2 is our study group (Nair/Bunt/Thiyya/Ezhava/Hoysala) of the South-west coastal India.

In the scatterplot, Nair shows higher Steppe and Iran_N related gene flow outcompeting all other South Asian groups except groups from Pakistan, North West India, and a few populations, including Bhumihar_Bihar and Rajput from Haryana (supplementary fig. S2a, Supplementary Material online). Interestingly, Nair displayed comparatively more gene flow from Iran_N than Cochin Jews and other populations of South-west coast or Godavari basin (Reddy and Vaidik Brahmins). Bunt and Hoysala exhibit similar trend as Nairs (supplementary fig. S2c and e, Supplementary Material online) but Thiyya and Ezhava show comparatively more shifting of other Indo-European groups toward right side of scatter, so more groups are outcompeting Thiyya and Ezhava in terms of gene flow from both sources viz. Steppe and Iran_N (supplementary fig. S2b and d, Supplementary Material online).

In the Maximum likelihood (ML) tree constructed using TreeMix v.1.12 (Pickrell and Pritchard 2012), the placement of all 5 groups was consistent with their clustering in PCA analysis, with Nair and Hoysala among North Indian Indo-European caste groups, and Thiyya and Ezhava are more toward Dravidian cluster (supplementary fig. S3, Supplementary Material online). However, we did not observe any population-specific drift among the sample groups. (supplementary fig. S3, Supplementary Material online).

### Fine Scale Population Structure Using Haplotype-Based Approach

To gain a better understanding of population structure and haplotype sharing pattern of 5 population groups of South-west coast India with modern Eurasians, we used haplotype-based approach with ChromoPainter (Lawson et al. 2012) and fineSTRUCTURE (Lawson et al. 2012). The fineSTRUCTURE (Lawson et al. 2012) clustering divided all Indian samples into 2 major clades, 1 with North West Indian and Gangetic plain groups and other clade with South Indian groups. South Indian clade was further divided into 2 major subclades, where one of them keeps Bunt and Hoysala individuals together, while the other one is heterogenous clade comprising all remaining populations in different branches together (supplementary fig. S4a and b, Supplementary Material online). This cluster includes Nair, Thiyya, Ezhava, some individuals of Bunt and Hoysala, Godavari basin populations such as; Reddy, Vaidik Brahmins, and Naidu, and also Dravidian groups namely; Kuruba, Kunabi, and Kurchas from Karnataka and Kerala. Havik and Karnataka Brahmins are also in this cluster along with Hoysala, Bunt, Nair, and Thiyya (supplementary fig. S4a and b, Supplementary Material online).

### Ancient Ancestral Contribution to South-West Coastal Groups

**Fig. 2.**
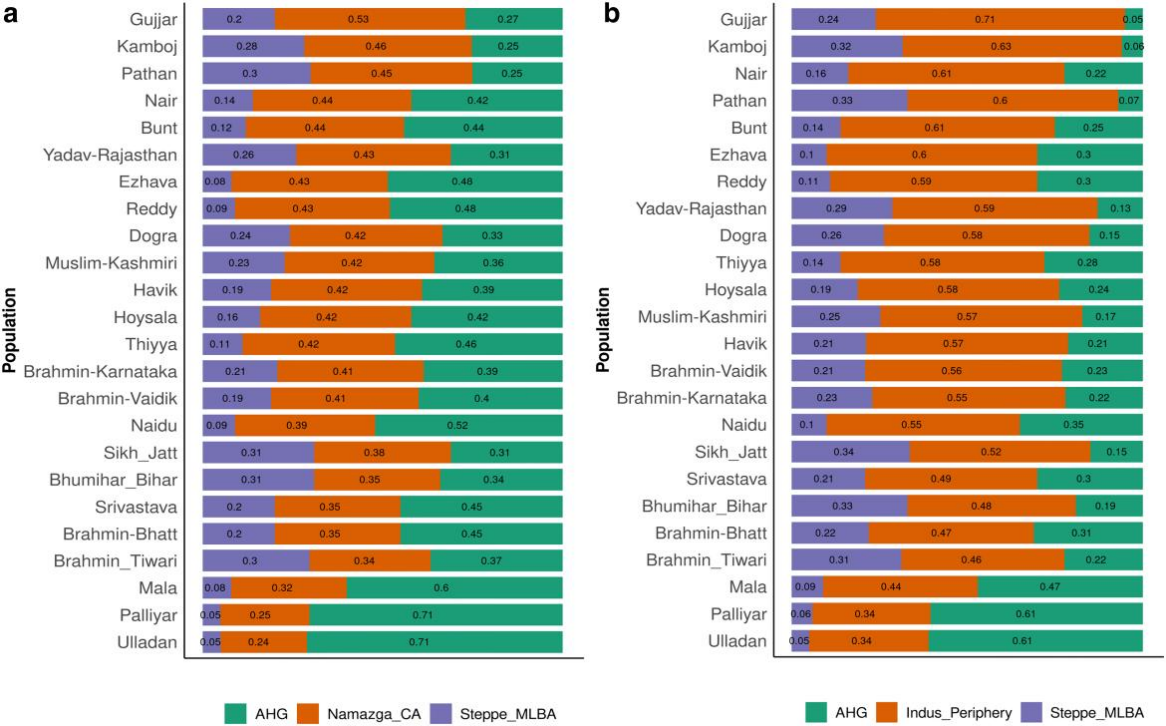
Admixture modeling to infer contribution of ancient ancestral west Eurasian source populations. a) Admixture modeling of South-west coastal groups along with some other modern Indian populations using AHG, Indus Periphery group and Middle and Late Bronze age Steppe groups (Steppe_MLBA) as ancient sources. Each color in bar plot represents fraction of ancestry from individual ancient source groups. b) Admixture modeling of South-west coastal groups using AHG, Namazga Chalcolithic group and Middle and Late Bronze age Steppe groups (Steppe_MLBA) as ancient sources.

We first tested the cladality of South-west coast populations with Gangatic plain (Brahmin_Tiwari, Bhumihar_UP) and also requirement of more than one source of ancestry using qpWave. We found that Brahmin_Tiwari and Bhumihar_UP forms clade with each other given the set of reference groups. After adding Nair or any other South-west coastal group qpWave model was satisfied with 2 distinct source of ancestry and not one source (supplementary table S10, Supplementary Material online). We further applied admixture modeling approach with 2 different sources of Iranian ancestry viz. pre-Bronze age Namazga_CA and Bronze age Indus_Periphery group using qpAdm of ADMIXTOOLS 2 to compare the ancient contributions into ancestry of 5 groups of South-west coast and other South Asian populations. In the first approach, we used Andamanese Hunter-Gatherers (AHG), Namazga_CA, and Steppe_MLBA as left groups. Among all South Asian groups tested, Namazga_CA component was comparatively higher in proportion in North West Indian groups, such as Gujjar (0.53), Kamboj (0.46), and Pathan (0.45) population from Pakistan. Surprisingly, all our 5 groups have higher proportion of Namazga_CA ancestry (Fig. 2a) (supplementary table S7, Supplementary Material online) along with other North West Indian groups, such as Muslim_Kashmiri, Dogra and Yadav_Rajasthan in comparison to Gangetic plain populations like Brahmin_Tiwari (0.34), Bhumihar_Bihar (0.35), and Srivastava (0.35) and also other Dravidians like Mala (0.32), Naidu (0.39), Palliyar (0.25), and Ulladan (0.24) from South India.

In the admixture modeling with Bronze age sources (AHG, Indus_Periphery, and Western/Central Steppe MLBA), we found that after Gujjar (0.71) and Kamboj (0.63), Nairs (0.61) are the group with higher Indus periphery component from Bronze age (Fig. 2b) (supplementary table S8, Supplementary Material online). Bunt is with similar contribution from Indus periphery group (0.44) and also Thiyya, Ezhava, and Hoysala along with groups from North West India like Yadav_Rajasthan, Dogra, and Muslim_Kashmiri are higher in distribution of Indus periphery component than most of the Gangetic Plain populations and other Dravidian population in South India (Fig. 2b) (supplementary table S8, Supplementary Material online).

We further tested all 5 groups from South-west coast India to fit in the Admixture graph topology comprising both modern and ancient population groups, using qpGraph function in ADMIXTOOLS 2. We tested different alternate topologies for all 5 groups to arrive at best-fitted model with ML score (closer to zero) and here we are showing only those results having best fit. For Nairs, we obtained a graph topology with best fit (likelihood score 2.94125), showing a pattern of admixture typical of ANI-ASI admixture from an ASI group like Palliyar and an ancient ghost population ANI formed by admixture between Indus population and Yamnaya like Steppe group (supplementary fig. S5a, Supplementary Material online). In addition to this simple ANI-ASI admixture, Nairs also requires another source group for Middle East ancestry from Bactria-Margiana-Archeological-Complex (BMAC) (supplementary table S9, Supplementary Material online).

We used Brahmin_Tiwari population from Uttar Pradesh to represent the Gangetic Plain group, which was in recent study found to be highest in carrying Steppe component (Narasimhan et al. 2019). While Nair required additional Middle Eastern component, Brahmin Tiwari from Gangetic plain was best fitted without it in a simple ANI-ASI admixture model (supplementary fig. S5a, Supplementary Material online). We applied admixture modeling approach with other study groups and found that all of these groups from South-west coast India are best fitted in the same model of additional Middle Eastern component along with ANI-ASI admixture required by other Indo-Europeans and Dravidian castes and tribes (supplementary fig. S5b to e, Supplementary Material online). Likelihood scores for Thiyya (3.294107), Bunt (2.919086), Ezhava (2.817161), and Hoysala (1.7) were maximum and closer to Zero value.

We also estimated the Admixture graph model for one of the North West Indian group to compare them with Gangetic plain populations and our South-west coastal study population. In this case, we used Gujjar population, which showed the highest proportion of Iranian ancestry in admixture modeling with source groups Namazga_CA (0.53) and Indus Periphery group (0.71) compared to all other south Asian populations.

In admixture graph modeling, Gujjar population also was best fitted in a model, where they require additional Middle Eastern component (supplementary fig. S5f, Supplementary Material online) along with the basic model of ANI-ASI admixture (Moorjani et al. 2013). The likelihood value for this admixture model for Gujjar population (2.3073) was maximum and very closer to zero (supplementary fig. S5f, Supplementary Material online).

### Estimation of Effective Migration Surface

Effective migration surface is the visual representation of geographical population structure in terms of effective migration. This representation of population structure highlights potential regions of higher-than-average and lower-than-average historic gene flow (Petkova et al. 2016). We applied this method to our study groups from South-west coast of India along with reference populations on ANI-ASI cline. We included in this analysis mainly the population groups from Indo-European and Dravidian linguistic families and excluded groups from Tibeto-Burman and Austroasiatic linguistic families, since we were mainly interested in migration events related to ANI-ASI admixture.

This method uses pairwise genetic dissimilarity matrix calculated from genotype data and geographical coordinates of samples as raw input and derives the posterior distribution of effective migration and diversity rates using MCMC iterations. Here, we first used varying number of Demes viz. 150, 175, 200, 225, and 250 demes 5 times each to run MCMC iteration and found 200 demes as best fitted for combined sample set. We further proceeded with main MCMC algorithm using 200 demes and varying acceptance proportions for proposal distributions to arrive at best suitable proposal distributions. After this, by applying these conditions, we ran main MCMC algorithm for 5 million MCMC iterations, 1 million burn-in, and 10,000 sample iterations. Based on final posterior distribution of effective migration and diversity rate, we plotted the migration surface.

We observed that there are 5 distinct regions across India having higher than average migration rate (supplementary fig. S6a, Supplementary Material online). First region is in the North India (in Jammu & Kashmir) and is continuous with North west India. Second region is mid-Gangetic plain of Uttar Pradesh and Bihar. Third region is in Central India, which is continuous with some regions in further west and also north. Fourth and fifth regions are in the Godavari basin and Karnataka, respectively, which are almost continuous to each other and some nearby regions extend further to coastal Karnataka. This reflects continuity in gene flow pattern across this large region in South India.

We also observed many regions with lower-than-average migration rates. One such region is in North West India separating 2 regions of higher-than-average migration rates in North India (region 1) and Central India (region 3). This region makes clear boundary or obstacle in gene flow between these 2 regions (supplementary fig. S6a, Supplementary Material online).

Other such regions with higher migration rates are between mid Gangetic plain and Godavari basin region and between Karnataka and Central Indian region. Southernmost part of India has another region of lower-than-average migration rates along with minor continuous regions of east coastal India. supplementary fig. S6b, Supplementary Material online represents the convergence of main MCMC algorithm, while supplementary fig. S6c, Supplementary Material online represents observed versus fitted dissimilarities between demes.

### Temporal Dynamics of Effective Migration Rates

We further explored the geographical gene flow pattern or migration rates dynamics over passage of time. Using matrix of similarity of shared long segments of haplotypes, also referred to as Long Pairwise Coalescent Segments (lPCS), among individuals of populations and varying lengths of these shared segments gives time-dependent estimates of effective population size and migration rates, a method implemented in Migration and Population Surface Estimation (MAPS) (Al-Asadi et al. 2019). Further, using geographical coordinates of samples along with genetic data, we inferred the geospatial patterns of migration rates and population size changes with time.

For this purpose, we obtained the phase genotype data and Identity By Descent (IBD) segments using Beagle5.1 (Browning et al. 2018) and Refined-IBD (Browning and Browning 2013) tools, then using predefined pipeline we derived the matrix of IBD sharing among individuals. We used 3 different long Pairwise Segment of Coalscence (lPSC) segment, lengths windows viz. 1 to 5 cM and 5 to 10 cM which corresponds to genetic time frame of 90 generation and 22 generations ago from present. We used IBD sharing matrix and geographical coordinates of samples and obtained the posterior distribution of the parameters effective migration rates and effective population size using MCMC algorithm. Using 200 demes, we first obtained the best-fitted variance proposals so that all values lie between 10% to 40% range. Then, we ran the main algorithm 10 times using 5 million MCMC, 1 million Burn-in, and 10,000 sample iterations and inferred the posterior distribution of parameters of effective migration rates and population sizes.

#### Effective Migration Surface in 1 to 5 cM Pairwise Segment of Coalescence (PSC) Length Window

**Fig. 3.**
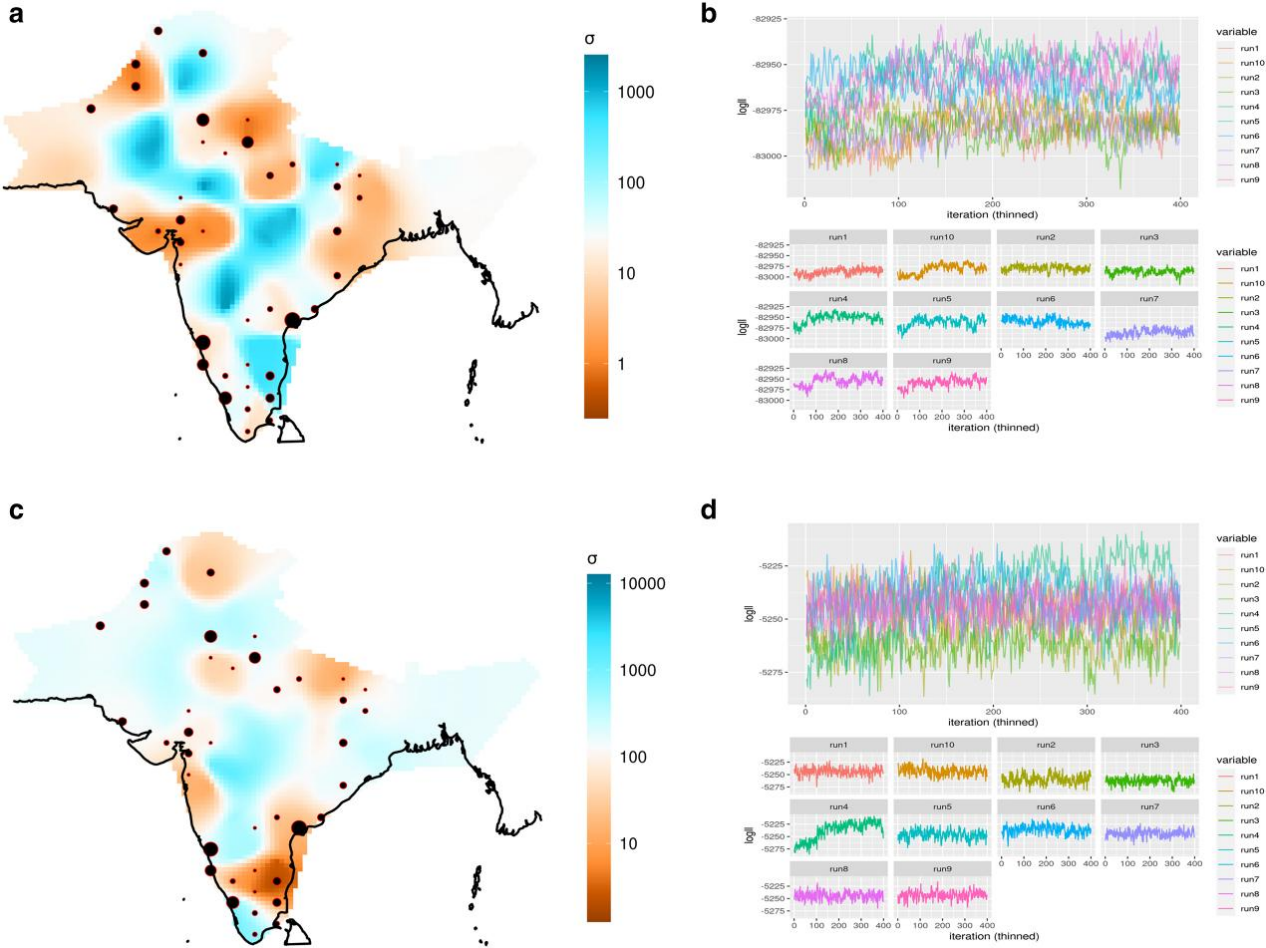
Spatial pattern of effective migration rates through time. a) Contour plot of effective migration rate in 1 to 5 cM lPSC length corresponding to a timeframe of 90 generations ago. Blue color represents regions of higher-than-average migration rates, while orange color shows regions of lower-than-average migration rates and b) corresponding MCMC iteration chain convergence. Plot shows the log likelihood distributions in 3 individual MCMC runs. c) Contour plot of effective migration rate in 5 to 10 cM lPSC length corresponding to a timeframe of 22 generations ago and d) corresponding MCMC iteration chain convergence in 3 individual runs.

In the Pairwise Segment of Coalescence (PSC) length segment range of 1 to 5 cM, which corresponds to mean coalescence time of 90 generation ago, we found that the North West India is having higher than average distribution of migration rate (Fig. 3a). Other regions include Central India and upper Godavari basin, and these 2 regions of higher-than-average migration rates are almost continuous to each other without any boundary. Another region with very high migration rate is near South East coast of India, but this region is separated from Godavari region by a boundary with lower-than-average migration rate (Fig. 3a).

#### Effective Migration Surface in 5 to 10 cM PSC Length Window

The PSC length window of 5 to 10 cM corresponds to a mean coalescence time of 22 generations ago from present. The migration surface at this time shows a diffused pattern in the regions with higher-than-average migration rates, like North West India, Central India, and Godavari basin, while regions in Gangetic Plain have lower than average migration rates (Fig. 3c). Upper Godavari basin is still a region of high migration rate compared to all other parts of India. Regions of South East coast, which were earlier regions of very high migration rates now shows a different pattern and becomes a region of very low migration rates. An additional region with higher-than-average migration rates appears in Southern most part of India, which is secluded by a boundary of low migration rates (Fig. 3c). Regions of North India near Jammu and Kashmir have now lower than average migration rates along with one more such region near North West India. Parts of Central India, Godavari basin, and parts of Karnataka including coastal Karnataka are now with similar migration rates (i.e. higher than average migration rates).

### Selection Scan on Genotype Data

We applied 3 different selection scans viz. PCAdapt (Luu et al. 2017), OutFlank (Whitlock and Lotterhos 2015), and GWDSFisher implemented in R package SambaR (de Jong et al. 2021). We found a total of 49 loci under positive selection in South-west coastal groups when compared to French population. Out of these 49 loci, 5 loci were common in all the 3 selection scans, which includes 3 contiguous loci (rs1257027, rs1730122, and rs1257017) on chromosome 2 (rfisherGWDS_log_p = 7.95; OutFLANK_log_p = 8.3), one (rs1133238) on chromosome 16 (rfisherGWDS_log_p = 7.97; OutFLANK_log_p = 8.18), and another (rs78832170) on chromosome 20 (rfisherGWDS_log_p = 8.07; OutFLANK_log_p = 7.99). Putative genes around 250 kb region of these 5 candidate loci were determined using bedtools2 (Quinlan and Hall 2010) *closestbed* command. Immunity-related genes (*TMEM131*, *ZAP70*, and *TRIM43*) were main putative gene signatures near candidate loci on chromosome 2. Genetic features near candidate loci on chromosome 16 were *SEPHS2* (related to Selenocysteine biosynthesis) and *ITGAL* (related to Immune response). While *WISP2* (related to Bone turnover), *KCNK15* (Potassium channel protein), and 2 immune related genes, *ADA* (T cell activation), and *SERINC3* (defense response to viruses) were main gene features near candidate region on chromosome 20.

### Mitochondrial Haplogroup Distribution Among South-West Coastal Groups

We compared the mtDNA haplogroup diversity among South-west coastal groups and found that maternal lineage is very diverse among Nair, Thiyya, and Bunt of Konkan and Malabar coast (supplementary fig. S9a, Supplementary Material online) (supplementary table S1, Supplementary Material online). We observed the prevalence of 5 major haplogroups (M, R, U, N, and H); of these, haplogroup M is the most prevalent followed by U and R. Surprisingly, we observed higher proportion of haplogroup H among Bunts of Konkan coast and with lesser frequency among Nair and Thiyya from Malabar (supplementary fig. S9a, Supplementary Material online) (supplementary table S1, Supplementary Material online). Haplogroup N was present in a significant fraction of Thiyya population, while haplogroup U had with highest occurrence among Nairs.

Among the M sub haplogroup, M3, M35, and M5 were present with highest frequency among Nair (M3 = ∼0.14), Thiyya (M35 = ∼0.12), and Bunt (M5 = ∼0.12) (supplementary fig. S9b, Supplementary Material online) (supplementary table S1, Supplementary Material online). The oldest sub haplogroup M2 was observed with noticeable frequency in all the 3 groups, while haplogroups M39 and M81 were only present in Bunt (M39 = 0.06; M81 = 0.01) and Thiyya (M39 = 0.01; M81 = 0.01). Some haplogroups like M57a and M64 (earlier observed among linguistic isolate Nihali population) are only observed in Thiyya while haplogroups M30, M61, M65b, and M80 were present only among Bunts. Among the R sub haplogroup, R6 and R7a were present in significant frequency among Nair (R6 = ∼0.08; R7a = ∼0.04) and Thiyya (R6 = ∼0.05; R7a = ∼0.03) while basal group R was found with frequency of 0.02 and 0.067 in Nair and Thiyya, respectively (supplementary fig. S9d, Supplementary Material online) (supplementary table S1, Supplementary Material online). Haplogroup R30 was present in Nair and Bunt, R5a in Bunt and Thiyya while R8 was found exclusively among Thiyya. Among the U sub haplogroup, U7a, U1a, and U2c were found among all 3 groups, while U2a, U2b, and U7b were present only in Nair and Thiyya. Haplogroup U5a was observed only in Bunt population while U9a was found among Bunt and Thiyya (supplementary fig. S9c, Supplementary Material online) (supplementary table S1, Supplementary Material online).

We also compared the mitochondrial DNA diversity among population using DnaSP. The observed haplotype diversity was comparable among Nair (0.99922 ± 0.0041), Bunt (0.99856 ± 0.00324), Thiyya (0.99949 ± 0.00295), Indian Indo-Europeans (0.99597 ± 0.0022), and Dravidian speakers (0.98861 ± 0.00498) (supplementary table S1a, Supplementary Material online). The nucleotide diversity within population was highest in Bunt (0.0018), compared to Nair (0.0015), and Thiyya (0.0014) but was lower than Indian Indo-Europeans (0.0021) (supplementary table S1a, Supplementary Material online).

## Discussion

South-west coastal India is a region of high population diversity and with complex genetic history. Some of the earlier studies on Jews (Behar et al. 2008, 2010; Chaubey et al. 2016), Parsees (Chaubey et al. 2017), and Roman Catholic (Kumar et al. 2021) have pointed out the complex genetic history and multilayered genetic structure of populations in this region. A few studies (Moorjani et al. 2013; Narasimhan et al. 2019) indeed suggested about the multilayered admixture in the Indian subcontinent, specially among caste groups. In the present study, we explored addition layers of genetic admixture and migrations in caste groups (majorly traditional warriors and landlords) of South-west coastal India.

Historical records suggest 2 distinct hypotheses regarding the origin of South-west coastal groups of traditional warriors or landlords’ status. According to oral tradition and some earlier genetic studies (Mahal and Matsoukas 2018; Nair et al. 2011), Nairs and Ezhava have common origin from North West Indian populations, particularly Sikh_Jat, which in turn were historically related to Indo-Scythian tribes (Mahil 1955; Dhillon 1994; Nijjar 2008; Marshall 2013). Some historians relate their origin to Iron Age populations of Ahichhatra, Uttar Pradesh (Fuller 1976). The outcome of our PCA analysis with autosomal dataset initially does not seem to be supporting any of the hypothesis, as populations like Nairs, Bunts, and Hoysala from South-west coast are clustering near North and North West Indians, but this may reflect higher ANI-ASI ratio compared to other Dravidian groups. Although the Placement of Thiyya and Ezhava more away toward Dravidian clusters points to a higher level of local admixture in them. However, clear presence of additional Middle Eastern component (highly prominent among groups that includes Balochi, Pathan and Sindhi from Pakistan and Kamboj, Dogra, Gujjar, and Yadav_Rajasthan from North West India) in Admixture analysis and also in our admixture modeling approaches contradicts the hypothesis of Ahichhatra origin of South-west coastal groups, while strengthens the hypothesis of their origin from a group closely related to North West Indian Indo-Europeans. Our Admixture F3 statistics also support this view, as Nair and Thiyya have highest affinity with Middle Eastern population rather than populations from Caucasus and Europe. Although Ezhava from Malabar, Reddy, Vaidik Brahmin from Godavari basin, and Gaud from Karnataka have this Middle Eastern component, but in lesser proportions. These groups possibly derived this additional component from ancestral groups of South-west coastal population while migration through Godavari basin and Karnataka, as our geospatial and temporal population structure analysis using EEMS and MAPS, suggests these regions along with Narmada basin to be key transition zone of gene flow from North West/North India to South India. Another plausible explanation here can be shared origin from a common ancestral lineage of Reddy from Godavari basin and Gaud from Karnataka with that of the populations of South-west coast. None of the group showed significant F3 statistics with Gangetic plain Indo-Europeans, but it was significant with North Western groups such as Kamboj, Gujjar, and Yadav_Rajasthan only in case of Nair, Bunt, and Thiyya. Admixture graph modeling approach also supports the presence of additional Middle Eastern component compared to Gangetic plain populations like Brahmin_Tiwari. The same graph model was also applicable to North West Indian groups like Gujjar. These results further strengthen the hypothesis of common origin of Nair, Bunt and Thiyya from a population related to those of modern North West Indian groups but definitely not from Indo-Europeans of Gangetic plain or the region near Ahichhetra (Uttar Pradesh) (Fuller 1976). Although this is well known that adding a greater number of admixture edged leads to overfitting of admixture graph model and care should be taken to interpret such a modeling approach. However, while testing different graph models, we applied additional admixture edges to both Gangatic plain groups and South-west coastal groups and we have reported only those models with minimum fit score in automated graph exploration method (find_graph) of Admixtools2.

Our Chromopainter-FineSTRUCTURE analysis suggests that Bunt and Hoyasla share more haplotypes and there was recent admixture between these 2 groups, while Nair, Thiyya, Ezhava, and remaining individuals from Bunt and Hoysala share haplotypes with most of the groups from South-west coast like Havik, Brahmin_Karnataka, Kuruba, Kunabi, and Kurchas and also Godavari basin like Vaidik Brahmin, Reddy, and Naidu, reflecting long admixture history with these groups or groups related to them.

In terms of mitochondrial lineage spread South-west coastal populations exhibit very high diversity with presence of different sub lineages of macro haplogroups M, R, U, and also presence of West Eurasian haplogroups HV and H. Although subhaplogroups of M, R, and U were observed in earlier studies with Indian populations (Kivisild et al. 1999; Cordaux et al. 2003; Metspalu et al. 2004; Palanichamy et al. 2004; Thangaraj et al. 2006; Chandrasekar et al. 2009), haplogroup H was observed with very low frequency among the caste groups of South India (Kivisild et al. 1999; Palanichamy et al. 2004). We observed high frequency of H haplogroup in Bunt population and HV in Thiyya population. Presence of high maternal haplogroup diversity among these groups points to possible admixture of diverse maternal lineage in them, which may be true because of their history of practicing matriarchy.

To sum up, we found a distinct group of populations from South-west coastal India, who belong to traditional warriors or feudal lords’ status and have genomic signature of Middle Eastern component compared to Gangetic plain Indo-Europeans and other Dravidian castes and tribes. This signature is also typical of some North West Indian populations like Gujjar, Kamboj and Yadav-Rajasthan. PCA clustering and haplotype sharing pattern indicate early population separation and isolation of South-west coastal populations from other Indo-Europeans of North and North West India and more local admixture. Study of geographical population structure and time-dependent migration rates suggests region of Central India (Narmada basin) and Godavari basin to be a transition zone for gene flow from North West or North India to South-west India. High mitochondrial haplogroup diversity along with comparatively higher prevalence of West Eurasian mtDNA haplogroup hinting female-mediated admixture instead of a male-mediated admixture, which is typical of most of the Indo-European castes in India.

## Materials and Methods

### Sampling

Blood samples were collected from 213 individuals belonging to Nair, Thiyya, Bunt, Ezhava, and Hoysala populations inhabited in Konkan and Malabar regions of Karnataka and Kerala states in India (Fig. 1a). All the subjects included in this study were healthy and unrelated. Informed written consent was obtained from each participant. The project was carried out in accordance with the guidelines approved by the Institutional Ethical Committees of Centre for Cellular and Molecular Biology, Hyderabad, India.

### Genotyping and Quality Control

DNA was isolated from the blood using standard phenol and chloroform methods. We genotyped 76 samples on Affymetrix Axiome GW Human Origin Array for 633,994 SNPs as per the manufacturer's specifications. Whole mitochondrial genome of all samples was also Polymerase Chain Reaction amplified using a set of 24 markers and sequencing was done using Sanger sequencing method in ABI 3730 Automated DNA analyzer (Applied Biosystems, Foster City, USA). All mtDNA sequences were assembled with the revised Cambridge reference sequence (rCRS) (Andrews et al. 1999) using AutoAssembler. Variations observed were used to assign the haplogroup using Phylotree build 17 (van Oven and Kayser 2009) and Haplogrep2 (Weissensteiner et al. 2016). Mitochondrial DNA diversity was calculated in DnaSP tool (Rozas et al. 2017).

For autosomal analysis, we merged our dataset with published DNA dataset (Reich et al. 2009; Moorjani et al. 2013; Mallick et al. 2016; Nakatsuka et al. 2017) of modern individuals after filtering for missingness using Plink 1.9 (Purcell et al. 2007), and included only autosomal markers on 22 chromosomes having genotyping call rate > 99% and minor allele frequency > 1%. We further pruned dataset by removing individuals with first-degree and second-degree relatedness utilizing KING-robust (Manichaikul et al. 2010) feature implemented in Plink2 (Chang et al. 2015). After all the filtering, final merged dataset comprised 968 contemporary individuals covering 422,810 SNPs.

In order to minimize the effect of background Linkage Disequilibrium (LD) in PCA and ADMIXTURE like analysis, we further thinned the markers by removing SNPs in strong LD (r2 > 0.4, window of 200 SNPs, sliding window of 25 SNPs at a time) using Plink 1.9 (Purcell et al. 2007). For all the analyses with ancient DNA, we merged our samples with West Eurasian aDNA published datasets (Meyer et al. 2012; Raghavan et al. 2014; Allentoft et al. 2015; Haak et al. 2015; Mathieson et al. 2015; Broushaki et al. 2016; Lazaridis et al. 2016; Yang et al. 2017; Damgaard et al. 2018; de Barros Damgaard et al. 2018; Narasimhan et al. 2019) of 1,245 individuals which are relevant as reference for our population. In this aDNA merged dataset, we applied the missingness criteria of geno > 0.7, to include only those individuals covered for at least 70% of sites resulting into 1,026 individuals covered at 441,609 sites.

### Genome-Wide SNP Data Analyses

We first performed PCA on merged dataset of modern Eurasian using the *smartpca* package implemented in EIGENSOFT 7.2.1 (Patterson et al. 2006) with default settings. We plotted first 2 components to infer genetic variability. We ran the model-based clustering algorithm ADMIXTURE (Alexander et al. 2009) to infer ancestral genomic components in all 5 groups of South-west coastal population inferred by PCA analysis. Cross validation was run 25 times for 12 ancestral clusters (*K* = 3 to *K* = 14). Lowest Cross Validation error parameter was obtained at *K* = 3. Therefore, we are showing the result for K value of 3. We constructed a ML tree for our merged dataset of modern West Eurasian and South Asian populations using TreeMix v.1.12 (Pickrell and Pritchard 2012) with LD blocks of 500 SNPs grouped together and Onge as an outgroup.

We used ADMIXTOOLS 2 package in R to calculate Admixture F3 statistics and D-statistics and to perform admixture modeling using qpAdm and qpGraph implementation. For F3 statistics calculation and admixture graph, we used precalculated F2 statistics setting parameter maxmiss = 1, while for calculation of D-statistics and admixture modeling, we used genotype file directly and limited the number of populations each time.

We used F3 statistics in the form of F3 (X, Palliyar; South-west coast population), where X is any modern West Eurasian or South Asian population and Palliyar was used as a proxy for ASI ancestry. We calculated D-statistics (Patterson et al. 2012) in the form of Dstat (Nair/Bunt/Thiyya/Ezhava/Hoysala, X, Y; Yoruba) to infer the extent of gene flow into 5 population groups of South-west coastal India. Where X is other South-west coastal or Godavari region population and Y is North West India, North India, Pakistan, and other west Eurasian populations.

We further utilized haplotype-based approach implemented in ChromoPainter (Lawson et al. 2012) and fineSTRUCTURE (Lawson et al. 2012) to derive co-ancestry matrix and fine scale population clustering, respectively. We first phased our data with SHAPEIT4 (Delaneau et al. 2019) using default parameters. Chromosome painting was performed using ChromoPainter (Lawson et al. 2012), first by performing 10 Expectation-Maximization (EM) iterations with 5 randomly selected chromosomes with a subset individual to infer global mutation rate (µ) and switch rate parameters (Ne). Then, we ran the main algorithm with 22 chromosomes and included all the individuals to get coancestry matrix. This coancestry matrix was used by fineSTRUCTURE (Lawson et al. 2012) to derive clustering using a probability model by applying Markov chain Monte Carlo (MCMC) procedure and then inferring hierarchical tree by merging all clusters with least change in posterior probability. For the run, we used 500,000 burn-in iterations and 1,000,000 subsequent iterations and stored the results from every 10,000th iteration.

To visualize geographical population structure and diversity across India and compare it with South-west coastal India, we ran Estimation of Effective Migration surface (EEMS) (Petkova et al. 2016). For this, we first applied different number of demes ranging from 150 to 250 and tuned proposal variances such as those that were accepted 10% to 40% of times. For final run we chose 200 demes, with 10 million MCMC, 2 million burn-in, and 10,000 thinning iterations for the determination of posterior distribution of effective migration and effective diversity rates.

In order to gain further insight into temporal dynamics of effective migration and diversity rates, we used the tool MAPS (Al-Asadi et al. 2019), which uses IBD sharing matrix and different length segments of IBD to track the change in effective migration and diversity rates with time. We used 2 different lengths intervals viz. 1 to 5 cM and 5 to 10 cM which corresponds to 90 generations and 22 generations ago, respectively. We used 200 demes with 5 million MCMC, 1 million burn-in and 10,000 thinning iterations for the determination of posterior distribution of effective migration and effective diversity rates. Here, again we tuned proposal variances for the acceptance proportions of 10% to 40% times.

In the analysis of the merged dataset with ancient DNA references, we computed D-statistics in the form F4 (pop1, pop2, Steppe/Yamnaya, Yoruba) and F4 (pop1, pop2, Iran_N, Yoruba) to compare relative affinity of various modern Indian populations for Steppe versus Iranian ancestry in comparison to our study groups. Here, pop1 is various modern Indian cline groups and pop2 is our study group (Nair/Bunt/Thiyya/Ezhava/Hoysala) of the South-west coastal India.

We used *qpadm* function of R package ADMIXTOOLS 2 to estimate proportions of ancient ancestral components in a test population (Nair/Bunt/Thiyya/Ezhava/Hoysala) derived from a set of N source population groups having shared drift with a set of reference populations. We performed modeling of admixture using Pre-bronze age and bronze age sources of Iranian ancestry viz Namazga_CA and Indus_Periphery group, respectively. We used Ethiopia_4500BP_published.SG, Anatolia_N, Dai.DG, Russia_EHG, WEHG, Jordan_PPNB, Ganj_Dareh_N, Israel_Natufian as references. We also did qpWave modeling to test for cladality of South-west coastal groups with Gangatic plain groups and also to test for need of more than one ancestry source compared to Gangatic plain groups. We also obtained fitted admixture graph topology with *qpGraph* function In ADMIXTOOLS 2 (Maier et al. 2022) package for 5 study groups along with Reddy population from Godavari basin and also Gujjar population from North West India and made a comparison with Gangetic plain population, Tiwari Brahmins.

Selection analyses were performed using the R packages OutFLANK-0.2 (Whitlock and Lotterhos 2015), pcadapt-4.3.3 (Luu et al. 2017), and the “gwdsfisher” function of SambaR (de Jong et al. 2021). Putative candidate genes around loci of selection were determined using bedtools2 (Quinlan and Hall 2010).

## Supplementary Material

evad225_Supplementary_Data

## Data Availability

Data has been uploaded to the CCMB server http://tdb.ccmb.res.in/bic/database_pagelink.php?page=wcdata and will be made available for the researchers upon request to the corresponding authors. The 1000 Genomes Project, https://www.internationalgenome.org/home. PhyloTree, http://www.phylotree.org/.

## References

[evad225-B1] Al-Asadi H, Petkova D, Stephens M, Novembre J. Estimating recent migration and population-size surfaces. PLoS Genet. 2019:15(1):e1007908. 10.1371/journal.pgen.1007908. 30640906 PMC6347299

[evad225-B2] Alexander DH, Novembre J, Lange K. Fast model-based estimation of ancestry in unrelated individuals. Genome Res. 2009:19(9):1655–1664. 10.1101/gr.094052.109. 19648217 PMC2752134

[evad225-B3] Allentoft ME, Sikora M, Sjögren K-G, Rasmussen S, Rasmussen M, Stenderup J, Damgaard PB, Schroeder H, Ahlström T, Vinner L, et al Population genomics of Bronze Age Eurasia. Nature. 2015:522(7555):167–172. 10.1038/nature14507. 26062507

[evad225-B4] Andrews RM, Kubacka I, Chinnery PF, Lightowlers RN, Turnbull DM, Howell N. Reanalysis and revision of the Cambridge reference sequence for human mitochondrial DNA. Nat Genet. 1999:23(2):147. 10.1038/13779. 10508508

[evad225-B5] Behar DM, Metspalu E, Kivisild T, Rosset S, Tzur S, Hadid Y, Yudkovsky G, Rosengarten D, Pereira L, Amorim A, et al Counting the founders: the matrilineal genetic ancestry of the Jewish Diaspora. PLoS One. 2008:3(4):e2062. 10.1371/journal.pone.0002062. 18446216 PMC2323359

[evad225-B6] Behar DM, Yunusbayev B, Metspalu M, Metspalu E, Rosset S, Parik J, Rootsi S, Chaubey G, Kutuev I, Yudkovsky G, et al The genome-wide structure of the Jewish people. Nature. 2010:466(7303):238–242. 10.1038/nature09103. 20531471

[evad225-B7] Broushaki F, Thomas MG, Link V, López S, van Dorp L, Kirsanow K, Hofmanová Z, Diekmann Y, Cassidy LM, Díez-del-Molino D, et al Early neolithic genomes from the eastern Fertile Crescent. Science. 2016:353(6298):499–503. 10.1126/science.aaf7943. 27417496 PMC5113750

[evad225-B8] Browning BL, Browning SR. Improving the accuracy and efficiency of identity-by-descent detection in population data. Genetics. 2013:194(2):459–471. 10.1534/genetics.113.150029. 23535385 PMC3664855

[evad225-B9] Browning BL, Zhou Y, Browning SR. A one-penny imputed genome from next-generation reference panels. Am J Hum Genet. 2018:103(3):338–348. 10.1016/j.ajhg.2018.07.015. 30100085 PMC6128308

[evad225-B10] Chandrasekar A, Kumar S, Sreenath J, Sarkar BN, Urade BP, Mallick S, Bandopadhyay SS, Barua P, Barik SS, Basu D, et al Updating phylogeny of mitochondrial DNA macrohaplogroup m in India: dispersal of modern human in South Asian corridor. PLoS One. 2009:4(10):e7447. 10.1371/journal.pone.0007447. 19823670 PMC2757894

[evad225-B11] Chang CC, Chow CC, Tellier LCAM, Vattikuti S, Purcell SM, Lee JJ. Second-generation PLINK: rising to the challenge of larger and richer datasets. Gigascience. 2015:4(1):7. 10.1186/s13742-015-0047-8. 25722852 PMC4342193

[evad225-B12] Chaubey G, Ayub Q, Rai N, Prakash S, Mushrif-Tripathy V, Mezzavilla M, Pathak AK, Tamang R, Firasat S, Reidla M, et al Like sugar in milk”: reconstructing the genetic history of the Parsi population. Genome Biol. 2017:18(1):110. 10.1186/s13059-017-1244-9. 28615043 PMC5470188

[evad225-B13] Chaubey G, Singh M, Rai N, Kariappa M, Singh K, Singh A, Pratap SD, Tamang R, Selvi RD, Reddy AG, et al Genetic affinities of the Jewish populations of India. Sci Rep. 2016:6(1):19166. 10.1038/srep19166. 26759184 PMC4725824

[evad225-B14] Cordaux R, Saha N, Bentley GR, Aunger R, Sirajuddin SM, Stoneking M. Mitochondrial DNA analysis reveals diverse histories of tribal populations from India. Eur J Hum Genet. 2003:11(3):253–264. 10.1038/sj.ejhg.5200949. 12678055

[evad225-B15] Damgaard PB, Marchi N, Rasmussen S, Peyrot M, Renaud G, Korneliussen T, Moreno-Mayar JV, Pedersen MW, Goldberg A, Usmanova E, et al 137 ancient human genomes from across the Eurasian steppes. Nature. 2018:557(7705):369–374. 10.1038/s41586-018-0094-2. 29743675

[evad225-B16] de Barros Damgaard P, Martiniano R, Kamm J, Moreno-Mayar JV, Kroonen G, Peyrot M, Barjamovic G, Rasmussen S, Zacho C, Baimukhanov N, et al The first horse herders and the impact of early Bronze Age steppe expansions into Asia. Science. 2018:360(6396):eaar7711. 10.1126/science.aar7711. 29743352 PMC6748862

[evad225-B17] de Jong MJ, de Jong JF, Hoelzel AR, Janke A. Sambar: an R package for fast, easy and reproducible population-genetic analyses of biallelic SNP data sets. Mol Ecol Resour. 2021:21(4):1369–1379. 10.1111/1755-0998.13339. 33503314

[evad225-B18] Delaneau O, Zagury JF, Robinson MR, Marchini JL, Dermitzakis ET. Accurate, scalable and integrative haplotype estimation. Nat Commun. 2019:10(1):5436. 10.1038/s41467-019-13225-y. 31780650 PMC6882857

[evad225-B19] Dhillon BS . History and study of the Jats: with reference to Sikhs, Scythians, Alans, Sarmatians, Goths, and Jutes. Gloucester (ON): Beta Publishers Incorporated; 1994.

[evad225-B20] Fuller CJ . The Nayars Today. Cambridge (UK): Cambridge University Press; 1976.

[evad225-B21] Haak W, Lazaridis I, Patterson N, Rohland N, Mallick S, Llamas B, Brandt G, Nordenfelt S, Harney E, Stewardson K, et al Massive migration from the steppe was a source for Indo-European languages in Europe. Nature. 2015:522(7555):207–211. 10.1038/nature14317. 25731166 PMC5048219

[evad225-B22] Kivisild T, Bamshad MJ, Kaldma K, Metspalu M, Metspalu E, Reidla M, Laos S, Parik J, Watkins WS, Dixon ME, et al Deep common ancestry of Indian and western-Eurasian mitochondrial DNA lineages. Curr Biol. 1999:9(22):1331–1334. 10.1016/s0960-9822(00)80057-3. 10574762

[evad225-B23] Kumar L, Farias K, Prakash S, Mishra A, Mustak MS, Rai N, Thangaraj K. Dissecting the genetic history of the Roman Catholic populations of West Coast India. Hum Genet. 2021:140(10):1487–1498. 10.1007/s00439-021-02346-4. 34424406

[evad225-B24] Lawson DJ, Hellenthal G, Myers S, Falush D. Inference of population structure using dense haplotype data. PLoS Genet. 2012:8(1):e1002453. 10.1371/journal.pgen.1002453. 22291602 PMC3266881

[evad225-B25] Lazaridis I, Nadel D, Rollefson G, Merrett DC, Rohland N, Mallick S, Fernandes D, Novak M, Gamarra B, Sirak K, et al Genomic insights into the origin of farming in the ancient Near East. Nature. 2016:536(7617):419–424. 10.1038/nature19310. 27459054 PMC5003663

[evad225-B26] Luu K, Bazin E, Blum MG. Pcadapt: an R package to perform genome scans for selection based on principal component analysis. Mol Ecol Resour. 2017:17(1):67–77. 10.1111/1755-0998.12592. 27601374

[evad225-B27] Mahal DG, Matsoukas IG. The geographic origins of ethnic groups in the Indian subcontinent: exploring ancient footprints with Y-DNA haplogroups. Front Genet. 2018:9:4. 10.3389/fgene.2018.00004. 29410676 PMC5787057

[evad225-B28] Mahil US . 1955. Antiquity of Jat Race. Delhi: Atma Ram & Sons.

[evad225-B29] Maier R, Flegontov P, Flegontova O, Changmai P, Reich D. 2022. On the limits of fitting complex models of population history to genetic data. bioRxiv: 2022.2005.2008.491072. 10.1101/2022.05.08.491072, 9 May 2022, preprint: not peer reviewed. PMC10310323 37057893

[evad225-B30] Mallick S, Li H, Lipson M, Mathieson I, Gymrek M, Racimo F, Zhao M, Chennagiri N, Nordenfelt S, Tandon A, et al The Simons Genome Diversity Project: 300 genomes from 142 diverse populations. Nature. 2016:538(7624):201–206. 10.1038/nature18964. 27654912 PMC5161557

[evad225-B31] Mangalore BMP . Keralolpathi (Malayalam Edition): CreateSpace Independent Publishing Platform; 2018. ISBN-101722056924.

[evad225-B32] Manichaikul A, Mychaleckyj JC, Rich SS Daly K, Sale M, Chen W-M. Robust relationship inference in genome-wide association studies. Bioinformatics. 2010:26(22):2867–2873. 10.1093/bioinformatics/btq559. 20926424 PMC3025716

[evad225-B33] Marshall J . A guide to Taxila. Cambridge (UK): Cambridge University Press; 2013.

[evad225-B34] Mathieson I, Lazaridis I, Rohland N, Mallick S, Patterson N, Roodenberg SA, Harney E, Stewardson K, Fernandes D, Novak M, et al Genome-wide patterns of selection in 230 ancient Eurasians. Nature. 2015:528(7583):499–503. 10.1038/nature16152. 26595274 PMC4918750

[evad225-B35] Metspalu M, Kivisild T, Metspalu E, Parik J, Hudjashov G, Kaldma K, Serk P, Karmin M, Behar DM, Gilbert MTP, et al Most of the extant mtDNA boundaries in south and southwest Asia were likely shaped during the initial settlement of Eurasia by anatomically modern humans. BMC Genet. 2004:5(1):26. 10.1186/1471-2156-5-26. 15339343 PMC516768

[evad225-B36] Meyer M, Kircher M, Gansauge M-T, Li H, Racimo F, Mallick S, Schraiber JG, Jay F, Prüfer K, de Filippo C, et al A high-coverage genome sequence from an archaic Denisovan individual. Science. 2012:338(6104):222–226. 10.1126/science.1224344. 22936568 PMC3617501

[evad225-B37] Moorjani P, Thangaraj K, Patterson N, Lipson M, Loh P-R, Govindaraj P, Berger B, Reich D, Singh L. Genetic evidence for recent population mixture in India. Am J Hum Genet. 2013:93(3):422–438. 10.1016/j.ajhg.2013.07.006. 23932107 PMC3769933

[evad225-B38] Nair SP, Geetha A, Jagannath C. Y-short tandem repeat haplotype and paternal lineage of the Ezhava population of Kerala, south India. Croat Med J. 2011:52(3):344–350. 10.3325/cmj.2011.52.344. 21674830 PMC3118723

[evad225-B39] Nakatsuka N, Moorjani P, Rai N, Sarkar B, Tandon A, Patterson N, Bhavani GS, Girisha KM, Mustak MS, Srinivasan S, et al The promise of discovering population-specific disease-associated genes in South Asia. Nat Genet. 2017:49(9):1403–1407. 10.1038/ng.3917. 28714977 PMC5675555

[evad225-B40] Narasimhan VM, Patterson N, Moorjani P, Rohland N, Bernardos R, Mallick S, Lazaridis I, Nakatsuka N, Olalde I, Lipson M, et al The formation of human populations in South and Central Asia. Science. 2019:365(6457):eaat7487. 10.1126/science.aat7487. 31488661 PMC6822619

[evad225-B41] Nijjar BS . Origins and history of Jats and other allied nomadic tribes of India: 900 BC-1947 AD. New Delhi (India): Atlantic Publishers & Dist; 2008.

[evad225-B42] Palanichamy MG, Sun C, Agrawal S, Bandelt H-J, Kong Q-P, Khan F, Wang C-Y, Chaudhuri TK, Palla V, Zhang Y-P. Phylogeny of mitochondrial DNA macrohaplogroup N in India, based on complete sequencing: implications for the peopling of South Asia. Am J Hum Genet. 2004:75(6):966–978. 10.1086/425871. 15467980 PMC1182158

[evad225-B43] Patterson N, Moorjani P, Luo Y, Mallick S, Rohland N et al Ancient admixture in human history. Genetics. 2012:192:1065–1093. 22960212 10.1534/genetics.112.145037 PMC3522152

[evad225-B44] Patterson N, Price AL, Reich D. Population structure and eigenanalysis. PLoS Genet. 2006:2(12):e190. 10.1371/journal.pgen.0020190. 17194218 PMC1713260

[evad225-B45] Petkova D, Novembre J, Stephens M. Visualizing spatial population structure with estimated effective migration surfaces. Nat Genet. 2016:48(1):94–100. 10.1038/ng.3464. 26642242 PMC4696895

[evad225-B46] Pickrell JK, Pritchard JK. Inference of population splits and mixtures from genome-wide allele frequency data. PLoS Genet. 2012:8(11):e1002967. 10.1371/journal.pgen.1002967. 23166502 PMC3499260

[evad225-B47] Pillai EK . Studies in Kerala history. Kottayam: National Book Stall; 1970.

[evad225-B48] Purcell S, Neale B, Todd-Brown K, Thomas L, Ferreira MAR, Bender D, Maller J, Sklar P, de Bakker PIW, Daly MJ, et al PLINK: a tool set for whole-genome association and population-based linkage analyses. Am J Hum Genet. 2007:81(3):559–575. 10.1086/519795. 17701901 PMC1950838

[evad225-B49] Quinlan AR, Hall IM. BEDTools: a flexible suite of utilities for comparing genomic features. Bioinformatics. 2010:26(6):841–842. 10.1093/bioinformatics/btq033. 20110278 PMC2832824

[evad225-B50] Raghavan M, Skoglund P, Graf KE, Metspalu M, Albrechtsen A, Moltke I, Rasmussen S, Stafford TW Jr, Orlando L, et al Upper Palaeolithic Siberian genome reveals dual ancestry of Native Americans. Nature. 2014:505(7481):87–91. 10.1038/nature12736. 24256729 PMC4105016

[evad225-B51] Reich D, Thangaraj K, Patterson N, Price AL, Singh L. Reconstructing Indian population history. Nature. 2009:461(7263):489–494. 10.1038/nature08365. 19779445 PMC2842210

[evad225-B52] Rozas J, Ferrer-Mata A, Sánchez-DelBarrio JC, Guirao-Rico S, Librado P, Ramos-Onsins SE, Sánchez-Gracia A. DnaSP 6: DNA sequence polymorphism analysis of large data sets. Mol Biol Evol. 2017:34(12):3299–3302. 10.1093/molbev/msx248. 29029172

[evad225-B53] Saletore BA . Ancient Karnataka. Poona: Oriental Book Agency; 1936.

[evad225-B54] Thangaraj K, Chaubey G, Singh VK, Vanniarajan A, Thanseem I, Reddy AG, Singh L. In situ origin of deep rooting lineages of mitochondrial Macrohaplogroup ‘M’ in India. BMC Genomics. 2006:7(1):151. 10.1186/1471-2164-7-151. 16776823 PMC1534032

[evad225-B55] Thomas R, Nair SB, Banerjee M. A crypto-Dravidian origin for the nontribal communities of south India based on human leukocyte antigen class I diversity. Tissue Antigens. 2006:68(3):225–234. 10.1111/j.1399-0039.2006.00652.x. 16948643

[evad225-B56] van Oven M, Kayser M. Updated comprehensive phylogenetic tree of global human mitochondrial DNA variation. Hum Mutat. 2009:30(2):E386–E394. 10.1002/humu.20921. 18853457

[evad225-B57] Weissensteiner H, Pacher D, Kloss-Brandstätter A, Forer L, Specht G, Bandelt H-J, Kronenberg F, Salas A, Schönherr S. HaploGrep 2: mitochondrial haplogroup classification in the era of high-throughput sequencing. Nucleic Acids Res. 2016:44(W1):W58–W63. 10.1093/nar/gkw233. 27084951 PMC4987869

[evad225-B58] Whitlock MC, Lotterhos KE. Reliable detection of loci responsible for local adaptation: inference of a null model through trimming the distribution of F(ST). Am Nat. 2015:186(Suppl 1):S24–S36. 10.1086/682949. 26656214

[evad225-B59] Yang MA, Gao X, Theunert C, Tong H, Aximu-Petri A, Nickel B, Slatkin M, Meyer M, Pääbo S, Kelso J, et al 40,000-year-old individual from Asia provides insight into early population structure in Eurasia. Curr Biol. 2017:27(20):3202–3208.e9. 10.1016/j.cub.2017.09.030. 29033327 PMC6592271

