# Supplementary material for: Genetic Affinities and Adaptation of the South-West Coast Populations of India
Source: Genome Biol Evol. 2023 Dec 11;15(12):evad225. doi: 10.1093/gbe/evad225 (PMC10745260; doi:10.1093/gbe/evad225)
Supplement: evad225_Supplementary_Data [file evad225_supplementary_data.zip › Supplementary_text.docx]

Supplementary material

**Genetic affinities and adaptation of the south-west coast populations of India**

Lomous Kumar^1^ · Anuhya Chowdhari^1^ · Jaison J. Sequeira^2^ · Mohammed S. Mustak^2^ · Moinak Banerjee^3^ · Kumarasamy Thangaraj^1^

^1^ CSIR-Centre for Cellular and Molecular Biology, Uppal Road, Hyderabad 500007, India

^2^ Department of Applied Zoology, Mangalore University, Mangalore, 574199, India

^3^Human Molecular Genetics Laboratory, Rajiv Gandhi Centre for Biotechnology, Thycaud PO, Thiruvananthapuram 695014, Kerala, India

**Brief Introduction and history of south west coastal populations**

**Nairs**

The Nayars were the prominent warriors or military class of Malabar region of India. Such a description is found in Pliny the Elder’s records (Natural History VI Chapter 2) (Holland and Club 1848). Socially, the Nayars ranked after the Nambuthiris in Malabar. And occupied same position in the Cochin and Travancore in Kerala. F. Fawcett in his book ‘Nayars of Malabar’ (1915) (Fawcett 1915) emphasises matriarchy followed in this population and states – “The chief immediate interest attached to them lies in the fact of their being the best, that is the fullest, the most complete existing example of matriarchy, or, to be more strictly accurate, of inheritance through females. This system, obtaining at one time amongst the Celts and other races of Europe, was probably universal in the sense that it existed at some period in the life history of mankind, and is now to be found here and there in the world”. He also goes on to speculate the origin of Nayars among the Aryan people as their physical features, rituals and life style are very different from the other Dravidians of the Madras Presidency. However, he concludes “It is not yet time to say whether they are or are not (Dravidians)”.

K. Ananthakrishna Iyer, in his book ‘Cochin Tribes and Castes’ (1912) (Iyer 1969), describes them as the last among the major prominent castes also referred to as the pure “Sudras of Malayala”. Nayars were mostly nobles and did not involve themselves in occupations carried out by the Tiyans or Ezhavas, such as handicrafts. They instead carried arms and believed to have descended from a noble lineage.

The word Nayar has origin from the word Nayaka, meaning groups which lead the people. K. A. Iyer also notes that some people relate Nayars to Nagas (snake worshipping Nagas or Scythians). Kerala Mahatmyan, a mythological and historical document in Sanskrit assumes Nayars to be originating from members of the Nambuthiri families and the Devagandharva and Rakshasa women brought in by Parasurama from extra-terrestrial regions. Another ancient record Keralotpatti trace their origin from the Sudras following the Brahman immigrants to Kerala. There are assumptions about their arrival from Tibet and Nepal. In the book ‘The Sun and the Serpent’ (1905) (Oldham 1905), C. F. Oldham tries to draw a link between the serpent worshippers of the north and south specifically between the Nairs and the Newars of Nepal. Not just the appearance and tradition, their languages also having an intimate connection with the Scythian or Turanean tongues. He goes on to conclude that the Nayars, the original serpent worshipping population belonged to the Naga colonists of the North when the Southern confines of Central Asia was dominated by Dravidian (Asuras) kingdoms. However, there is a general consensus of opinion that Nayars are early immigrants from the Tamil country.

The Nayars are divided into clans, mostly exogamous with some endogamous groups. F. Fawcett also notes that the Nayar women of North Malabar were prohibited from intermingling with the men of South Malabar whom they considered inferior. This restriction was followed by the South Malabar clans too. The Nayars followed an interesting tradition which describes the social structure of the Malabar in the earlier times. The soiled cloth of the Nayar woman during her menstruation, as a rule, was washed by the washerwoman of the Tiyan caste and one of her own clean clothes were given to her instead. This was considered a purification process known as ‘mattu’ (change). This word is still in use in neighbouring Kannada and Tulu languages.

Nayars/Nairs at present constitute to 15-20% of the total population in Kerala (Various Surveys) with noticeable representation in the fields of Art, Science and Entrepreneurship.

**Thiyya**

Thiyya/Tiyans is a toddy-drawing caste from of Malabar, Cochin and Travancore. Until the 19^th^ century this middle-class community was involved in any kind of work like cultivating the land, follow trades and professions, take service as domestics and so on. “Anything but soldiering, for which they have an utter abhorrence”, Edgar Thurston states in his anthropological book series namely ‘Castes and Tribes of Southern India’ Volume VII published in 19’ (Thurston and Rangachari 2018). During the British rule, there are official records stating European admixture which could be attributed to any Caucasian haplogroups found in the study.

It is believed that Tiyans and Izhuvans (another toddy drawing caste) migrated from Ceylon. The South Canara Manual (Sturrock 1894) states that “it is well known that both before and after the Christian era there were invasions and occupation on the northern part of Ceylon by the races then inhabiting Southern India, and Malabar tradition tells us that some of these Dravidians migrated again from Iram or Ceylon northwards to Travancore and other parts of the west coast of India, bringing with them the cocoanut or the southern tree (Tengina Mara) and being known as Tivars (Islanders) or Iravars, which names have been altered to Tiyars and Ilavars”.

Socially, Tiyars who were considered to be subordinates of Nayars and Nambutiris, wouldn’t prefer to have food prepared by an Izhuvan. There is restriction of marriage the same illam (father’s family). Each illam is exogamous. The main feature of the Thiyyan religion is that they are more inclined to Shakti worship. Arrack is prominently used in ceremonies instead of milk or honey. There are a lot of similarities between the cultural and social practices of Tiyans, Ilavas (Izhuvans), Billavas and Poojaris. Sri Narayan Guru is revered by the people of all these communities. It is to be noted that, these communities are matrilineal. However, A Shreedhar Menon in his ‘A survey of Kerala History’ (Menon 2006), opines that Marumakkattayam, matrilineal system was introduced into the Malabar region after the Chola Chera war during the eleventh century A.D. In his thesis titled ‘The History of the Awakening of the Ilava community in Kerala’ (Jacob 1982), George Jacob groups Tiyans as subset of the larger modern day Ilava community, limited to the North Malabar region. A. Aiyyappan in his book ‘Iravas and culture change’ (Aiyappan 1944) describes ‘Tiya’ as a synonym for Iravas/Ilav/Izhuvans in North Malabar, ‘Tandan’ in Walluvanad taluk of South Malabar and ‘Chovan’ in southern divisions in Cochin. He also states that since Tiyas are educationally and economically more advanced, at every census, Iravas and Tandans identify themselves as Tiyas.

The legends, however, have differentiated Tiyas and Iravas distinctly. Iravas originally came from Ceylon and settled in the Malabar region. An ancient king of Malabar insulted the artisan section of the population, who left his kingdom and settled in Ceylon. On their return they were escorted by the ‘protectors’ who the Iravas claim to be their ancestors. One of the well-known legends classifies Tiyas and Tandans as descendants of the returned Iravas from Ceylon and the local Brahman woman. While the Irava Pannikars descended from a Chruma woman.

**Bunts**

Bunt is a warrior class community in the coastal districts of Karnataka speaking Tulu and Kundagannada and were traditionally agricultural groups mainly involved in cultivation of Paddy. They follow a matrilineal system also known as Aliyasantana. There are 93 clans, which are further divided into 53 Bari (or matrilineal septs). There is strict marriage restriction between same Bari. According to description by Historian Edgar Thurston in his book "Caste and tribes of south India" (1909) - "Men and women of the Bunt community belong to a beautiful race of Asia. Men have a broad forehead and a parrot nose. Mostly they are of fair complexion. Even today they are of independent nature, short tempered, self-respecting and have a muscular body, which tells about the history of belonging to warrior families" (Thurston and Rangachari 2018).

Bunts relates their origin from an ancient dynasty Alupa (200 CE - 1500 CE) and according to Historian P. Gururaja Bhat the royal family of this dynasty were majorly belonging to the Bunt caste. According to Indian anthropologist Ayinapalli Aiyappan warrior clan of Kolathunadu (Kola Bari and the Kolathiri Raja) was a descendant of the Bunts. As mentioned by Norwegian anthropologist Harald Tambs-Lyche Jain kingdoms of Canara region had warriors from Bunt clans and also Bunts were appointed as military officers under The Hoysala Ballal kings.

**Ezhava**

Ezhava is a population of Malabar coast of India constituting around 23% of the population of the area. They are social groups of agricultural labourers, small cultivators, toddy tappers along with involvement in weaving and also practicing Ayurveda. According to one theory based on numerous shared customs like childbirth, death, their matrilineal practices and martial history, they share common heritage with the Nairs.

Ezhava also worked as warrior’s clan under different rulers of Kerala like Kadathanad and Kurumbranad of Kerala. A subgroup of the Ezhavas considered themselves to be warriors and became known as the Chekavars. The Vadakkan Pattukal ballads describe Chekavars as forming the militia of local chieftains and kings but the title was also given to experts of Kalari Payattu.

**Hoysala**

Hoysala are the prominent Brahmin communities of Karnataka. They mainly speak Kannada as their mother tongue, live in the Southern Districts of Karnataka. According to historians they have been natives of the Malnad region of Karnataka but inscriptions also point towards their contacts with Yadava from north India. During 10^th^ century to 14^th^ century, they ruled a large region of Karnataka, parts of Andhra Pradesh and Tamil Nadu as Hoysala dynasty. This dynasty was founded by King Nripa Kama ii, who was having an alliance with the western Ganga dynasty. Although proper records are lacking to link Hoysala to the Yadavas from north India.

**Supplementary Table 1**

| **Details of reference samples and corresponding geographical regions** | | | | | |
| --- | --- | --- | --- | --- | --- |
| **North/Northwest India** | | **Gangetic Plains** | | **Godavari Basin** | |
| **1** | Sikh Jatt | **1** | Brahmin Tiwari | **1** | Reddy Telangana |
| **2** | Brahmin Haryana | **2** | Bhumihar Bihar | **2** | Gaud |
| **3** | Yadav Rajasthan | **3** | Bhumihar Uttar Pradesh | **3** | Kapu Andhra Pradesh |
| **4** | Kamboj | **4** | Yadav Uttar Pradesh | **4** | Vaidik Brahmin |
| **5** | Gujjar | **5** | Lodhi | **5** | Madiga |
| **6** | Dogra | **6** | Srivastava |  |  |
| **7** | Rajput | **7** | Nai |  |  |
| **8** | Khatri | **8** | Baniya |  |  |
| **9** | Kashmiri Muslims | **9** | Dushadh |  |  |
|  |  | **10** | Kanjad |  |  |
|  |  | **11** | Kshatriya Durgvanshi |  |  |
|  |  | **12** | Kurmi UP |  |  |

**Supplementary Figures**

**Fig S1a-e. Admixture_F3_statistics of south west coastal populations.**

**A.** Admixture_F3_statistics as F3(Nair; Palliyar, X), where X is any modern west Eurasians. **B.** Admixture_F3_statistics as F3(Thiyya; Palliyar, X), where X is any modern west Eurasians. **C.** Admixture_F3_statistics as F3(Bunt; Palliyar, X), where X is any modern west Eurasians. **D.** Admixture_F3_statistics as F3(Ezhava; Palliyar, X), where X is any modern west Eurasians. **E.** Admixture_F3_statistics as F3(Hoyslal; Palliyar, X), where X is any modern west Eurasians.

A


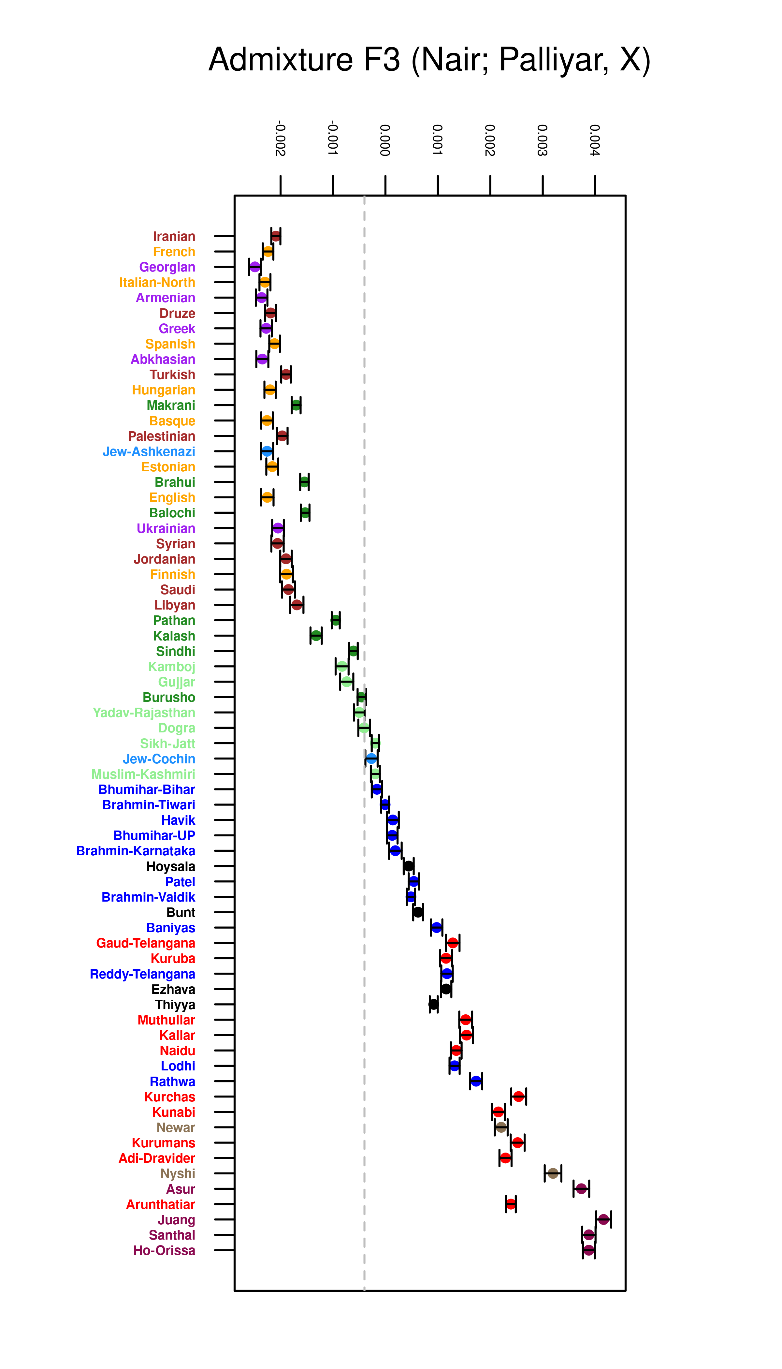


B


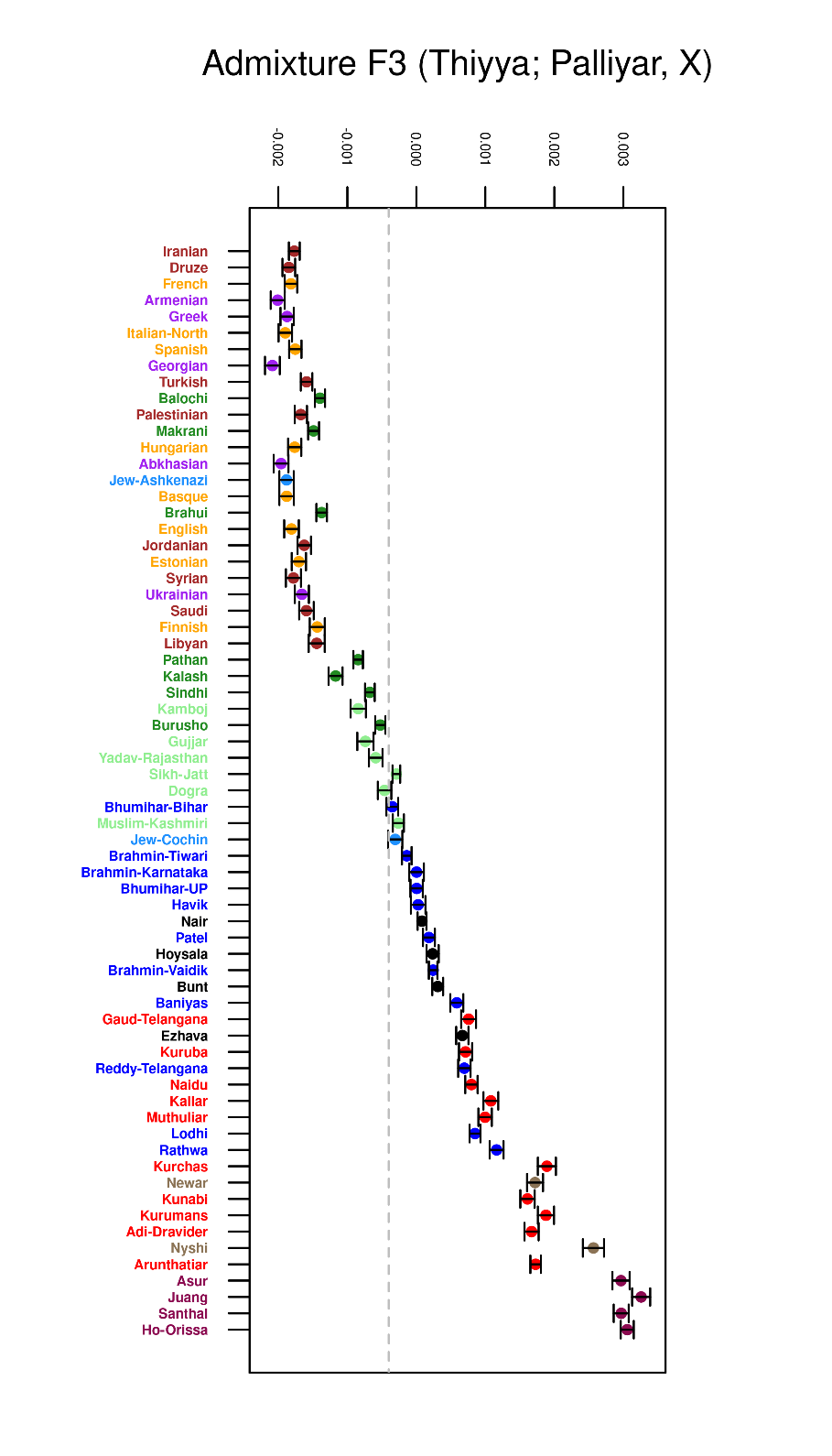


C


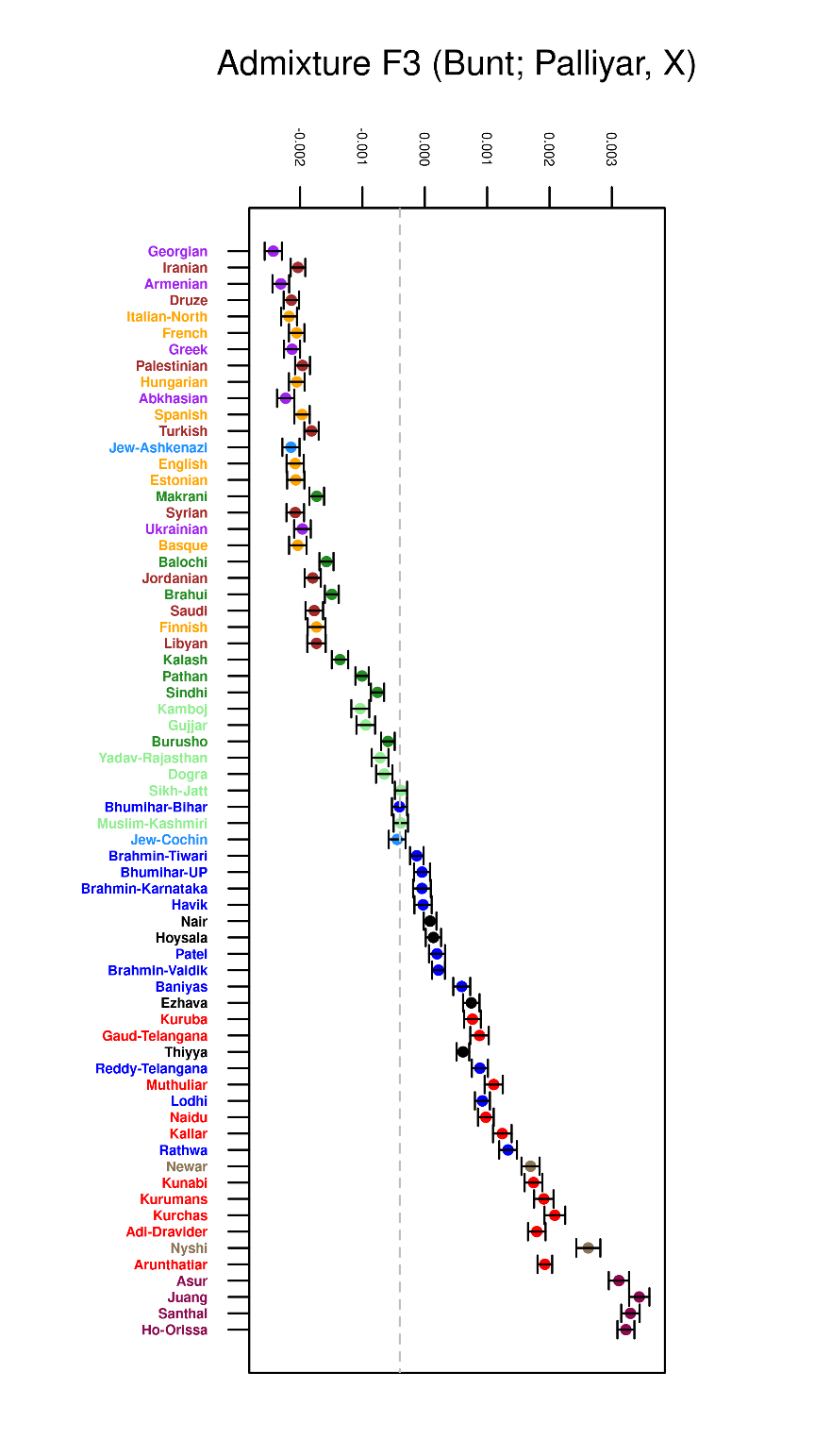


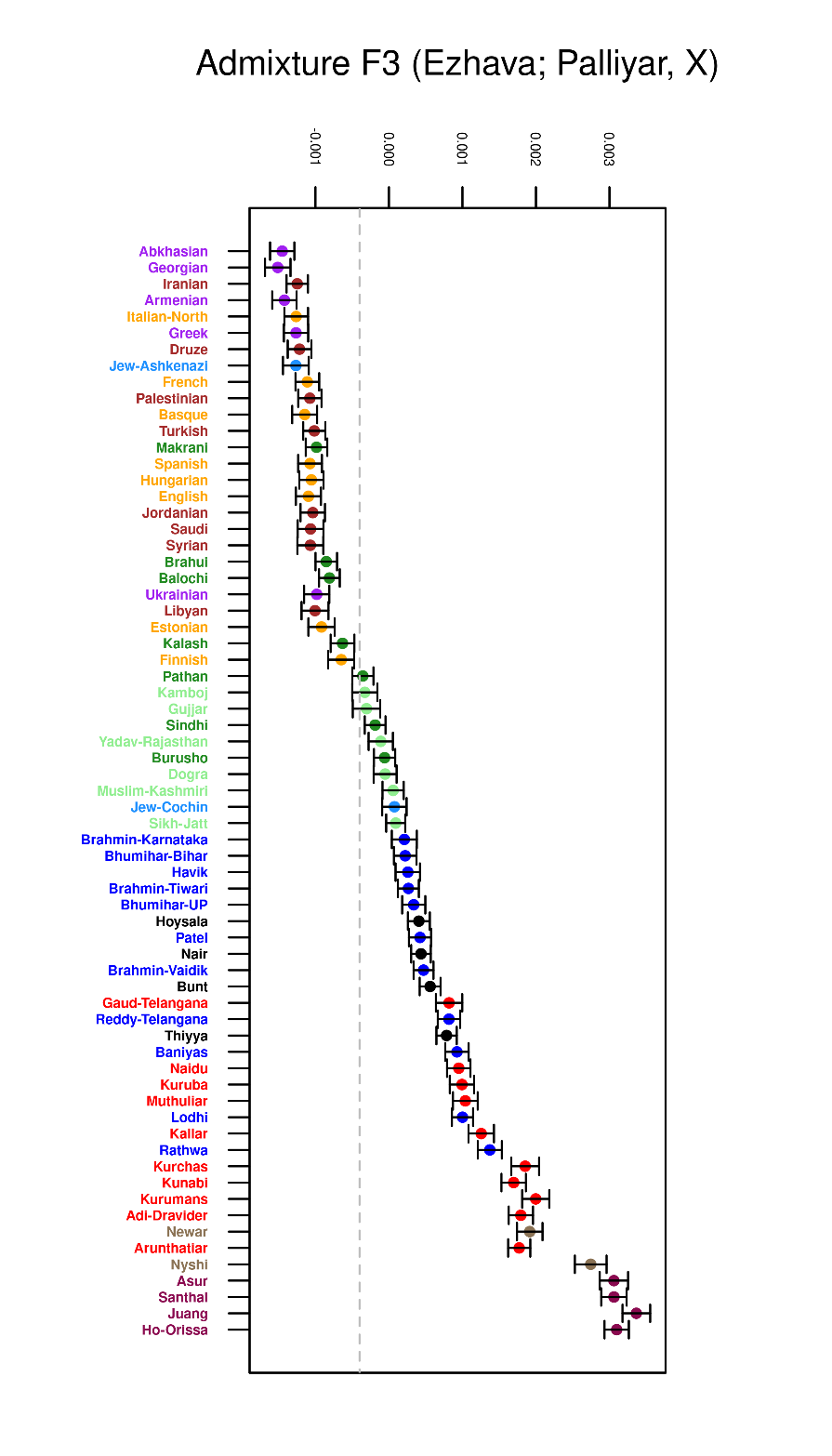


D

E


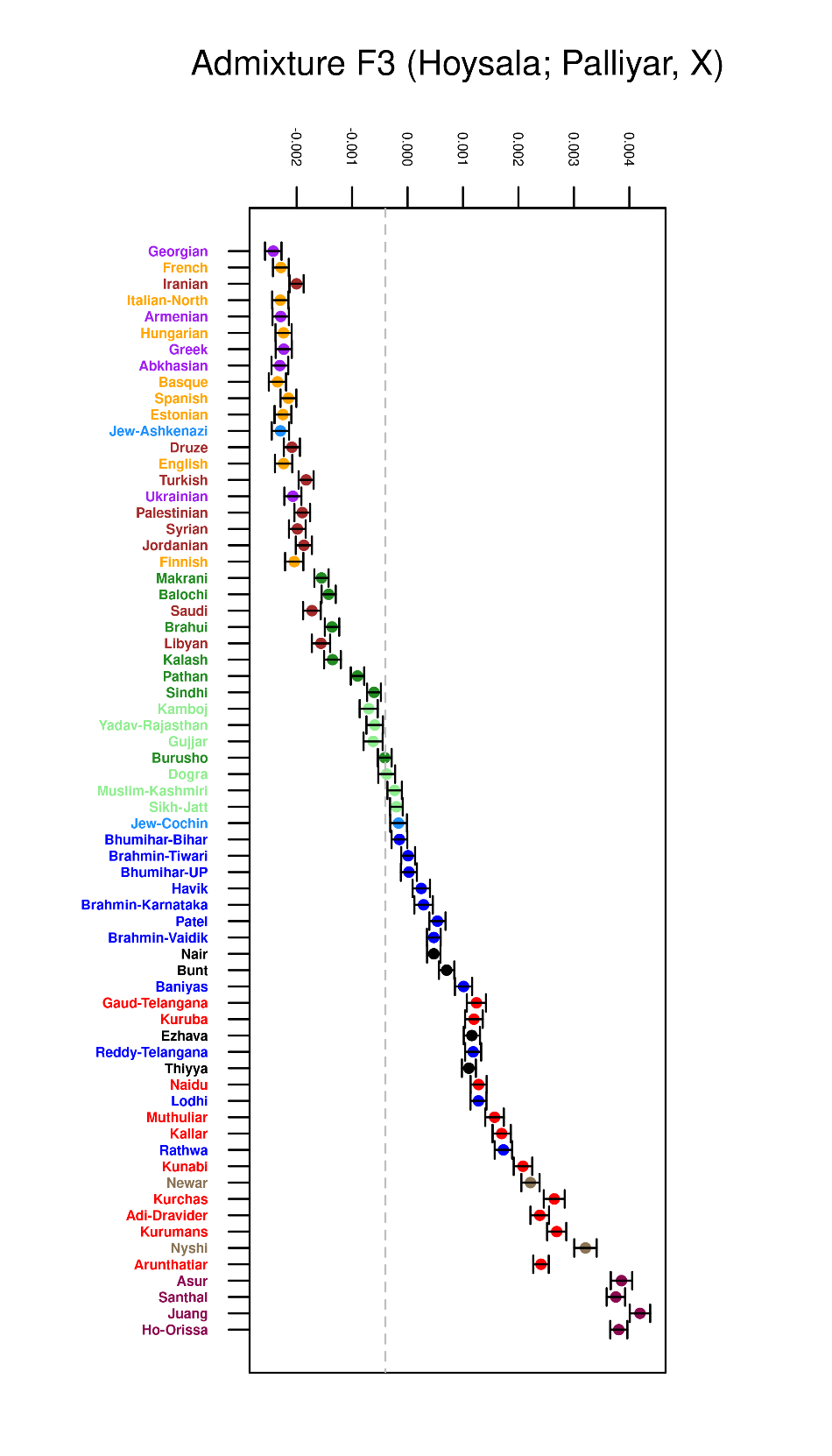


**Fig S2. *D* statistics of south west coastal population with ancient sources. A.** Steppe_EMBA vs. Iran_N scatterplot comparing D(X, Nair; Yamnaya, Yoruba) vs D(X, Nair; Iran_N, Yoruba), where X is different modern west Eurasian groups. **B.** Steppe_EMBA vs. Iran_N scatterplot comparing D(X, Thiyya; Yamnaya, Yoruba) vs D(X, Thiyya; Iran_N, Yoruba). **C.** Steppe_EMBA vs. Iran_N scatterplot comparing D(X, Bunt; Yamnaya, Yoruba) vs D(X, Bunt; Iran_N, Yoruba). **D.** Steppe_EMBA vs. Iran_N scatterplot comparing D(X, Ezhava; Yamnaya, Yoruba) vs D(X, Ezhava; Iran_N, Yoruba). **E.** Steppe_EMBA vs. Iran_N scatterplot comparing D(X, Hoysala; Yamnaya, Yoruba) vs D(X, Hoysala; Iran_N, Yoruba).


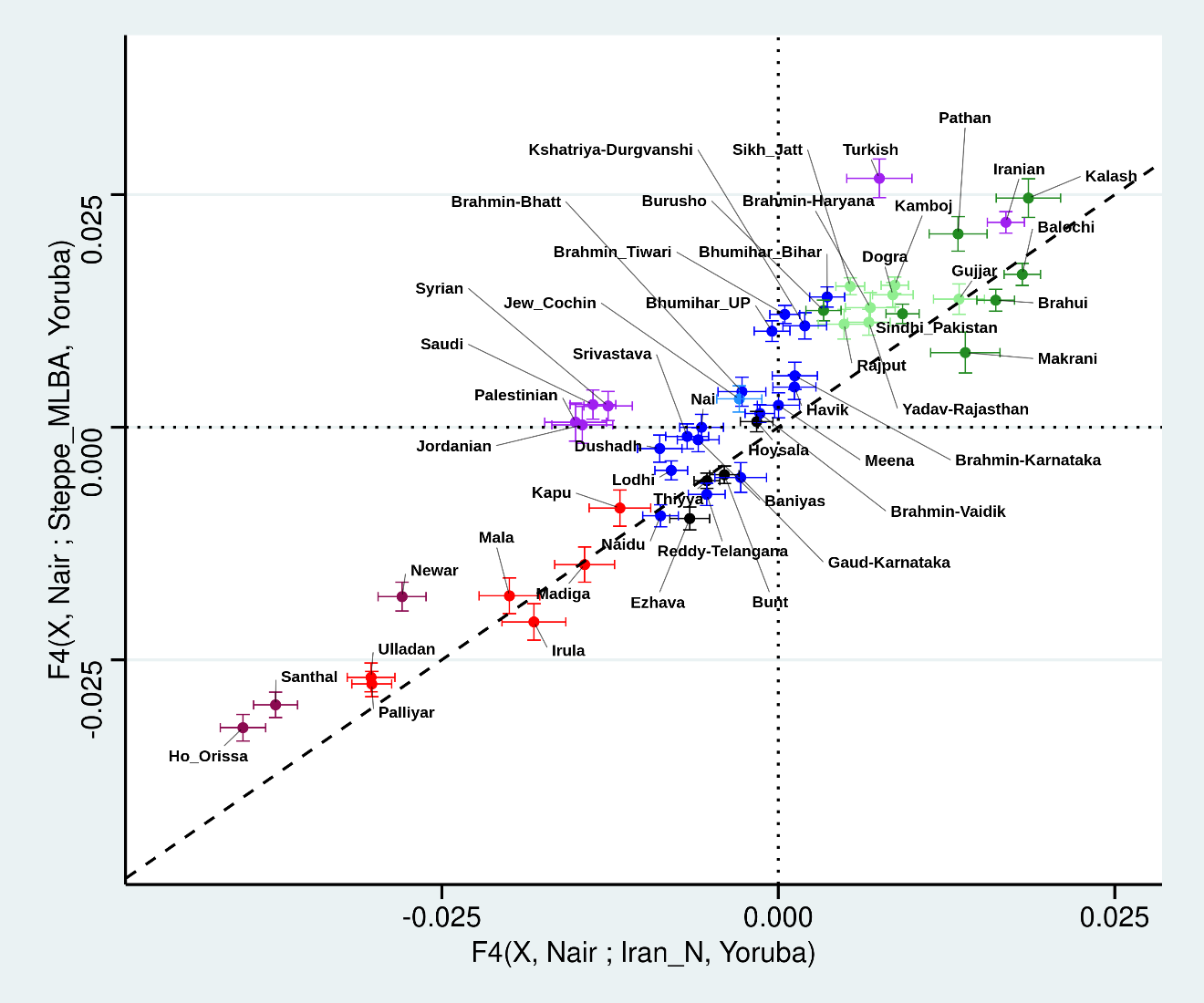


A


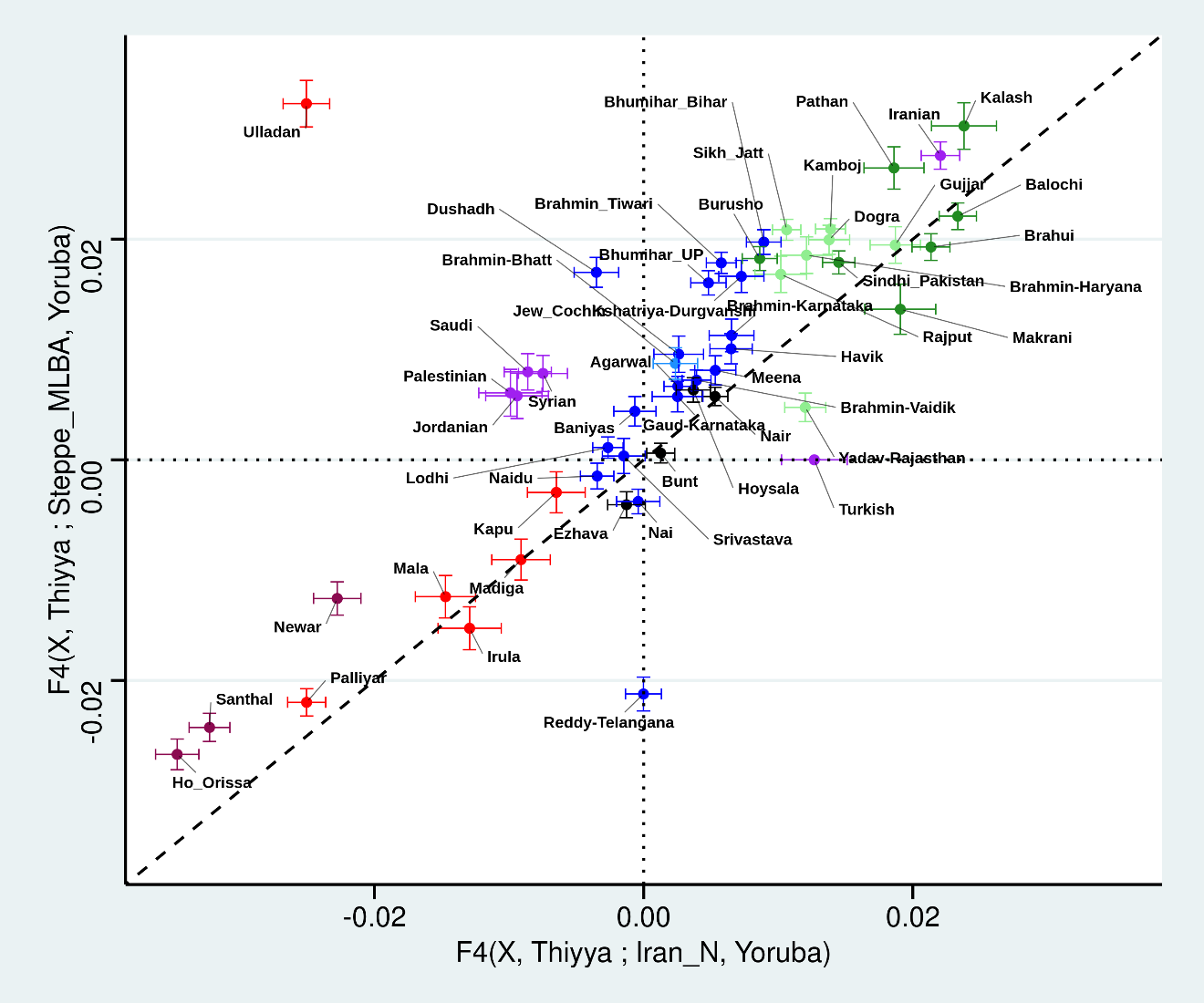


B

C


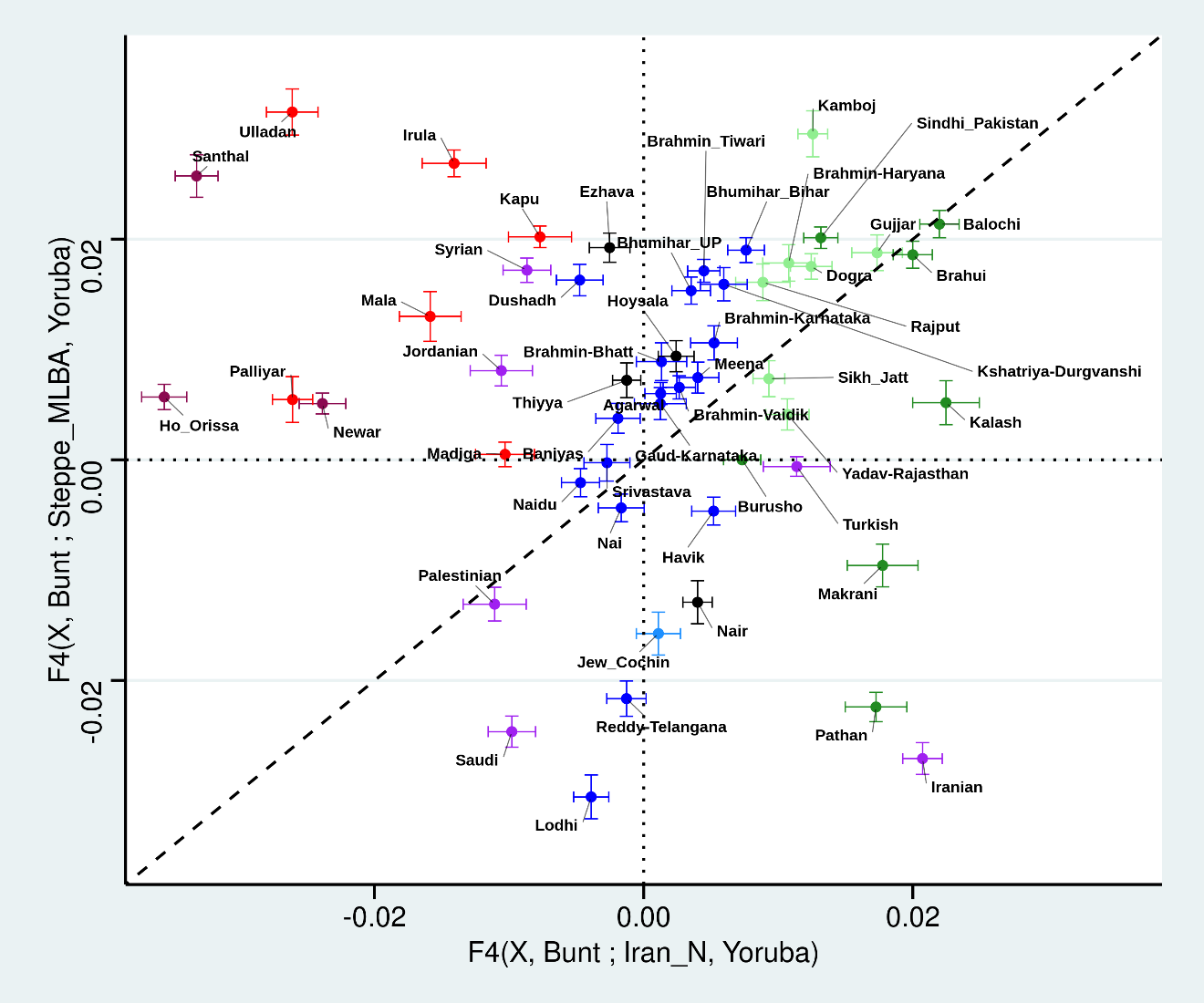


D


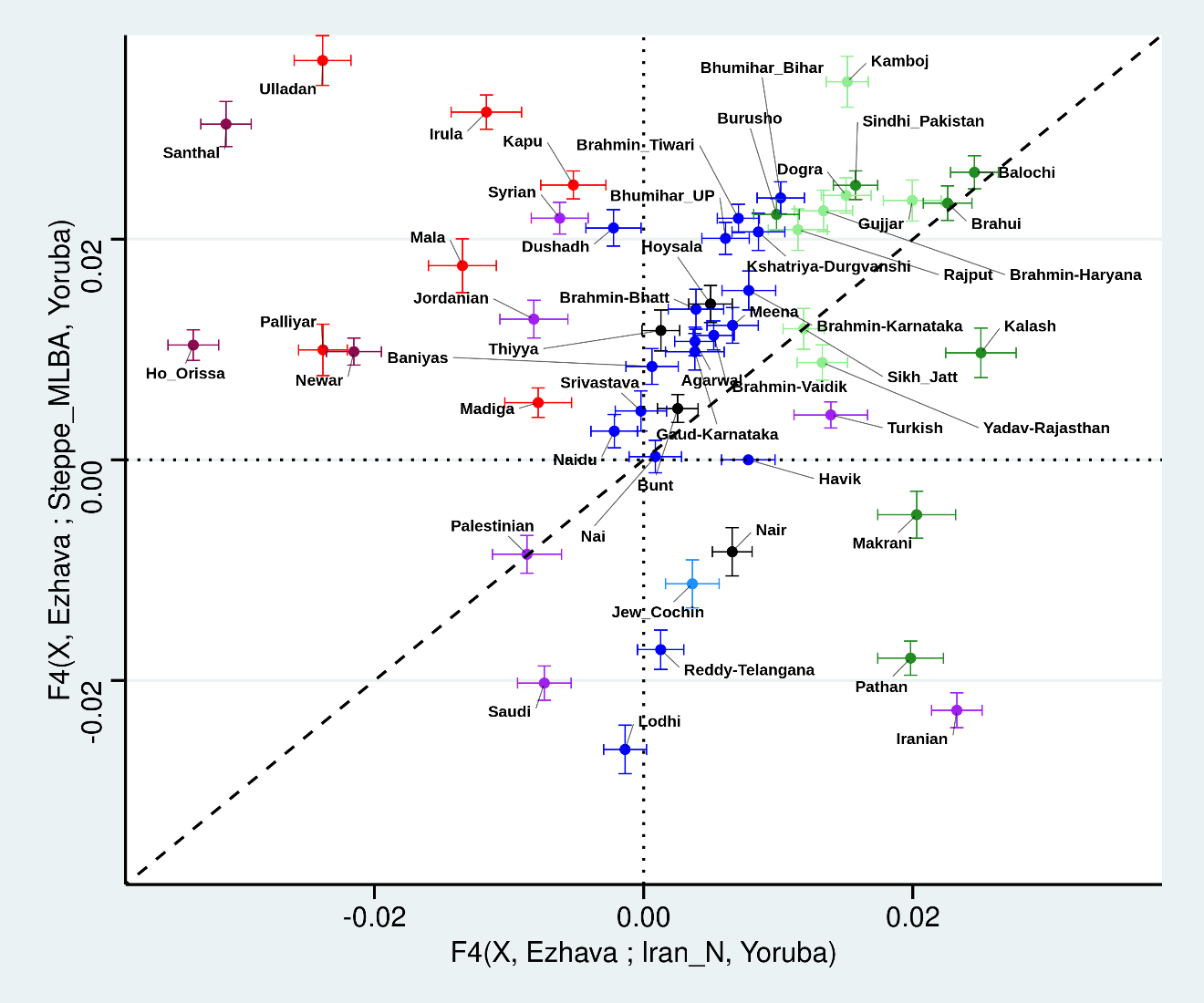


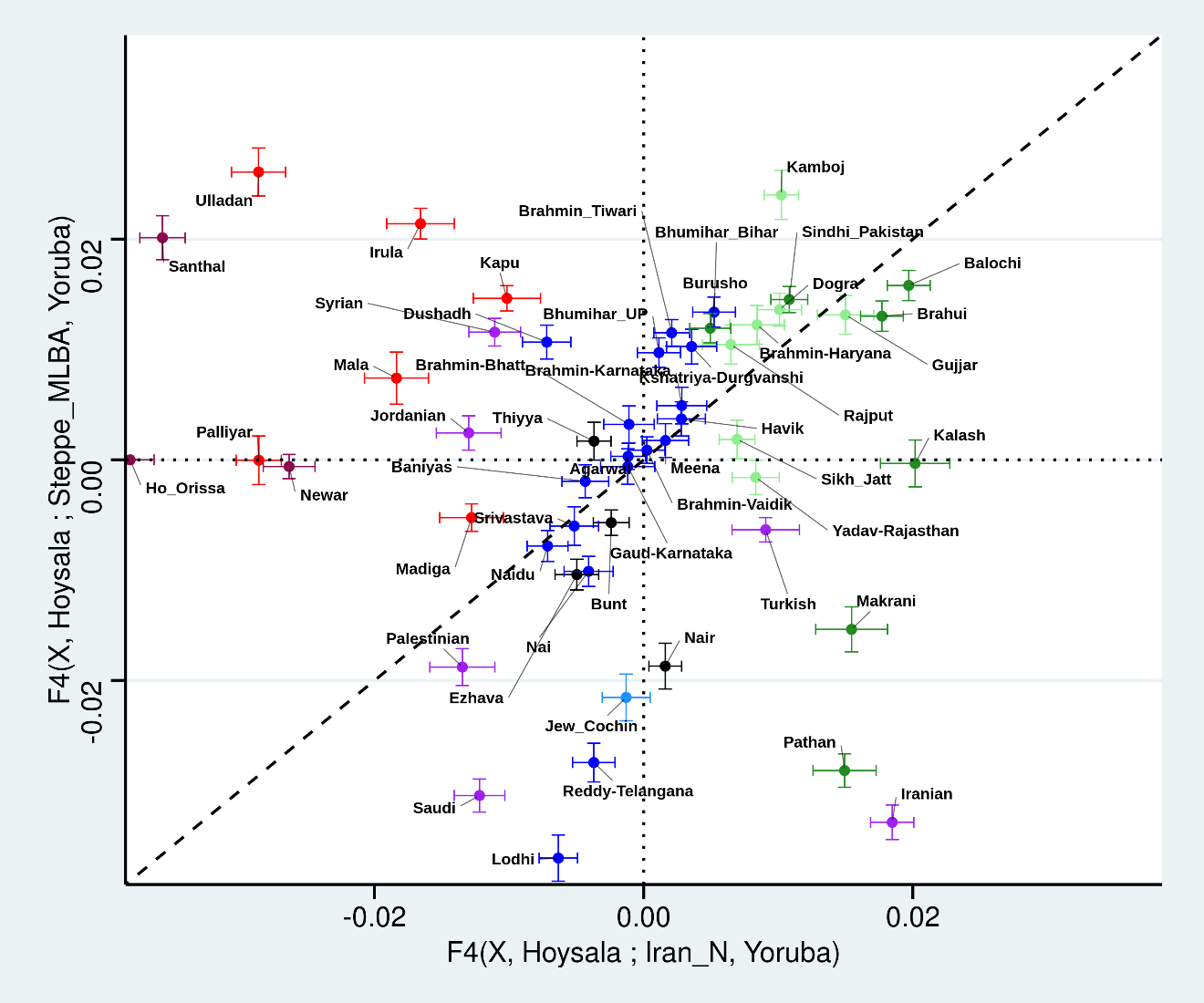


E

**Fig S3. Maximum Likelihood tree using TreeMix**: relationship of south west

coastal groups with other south Asian and west Eurasians inferred by TreeMix.


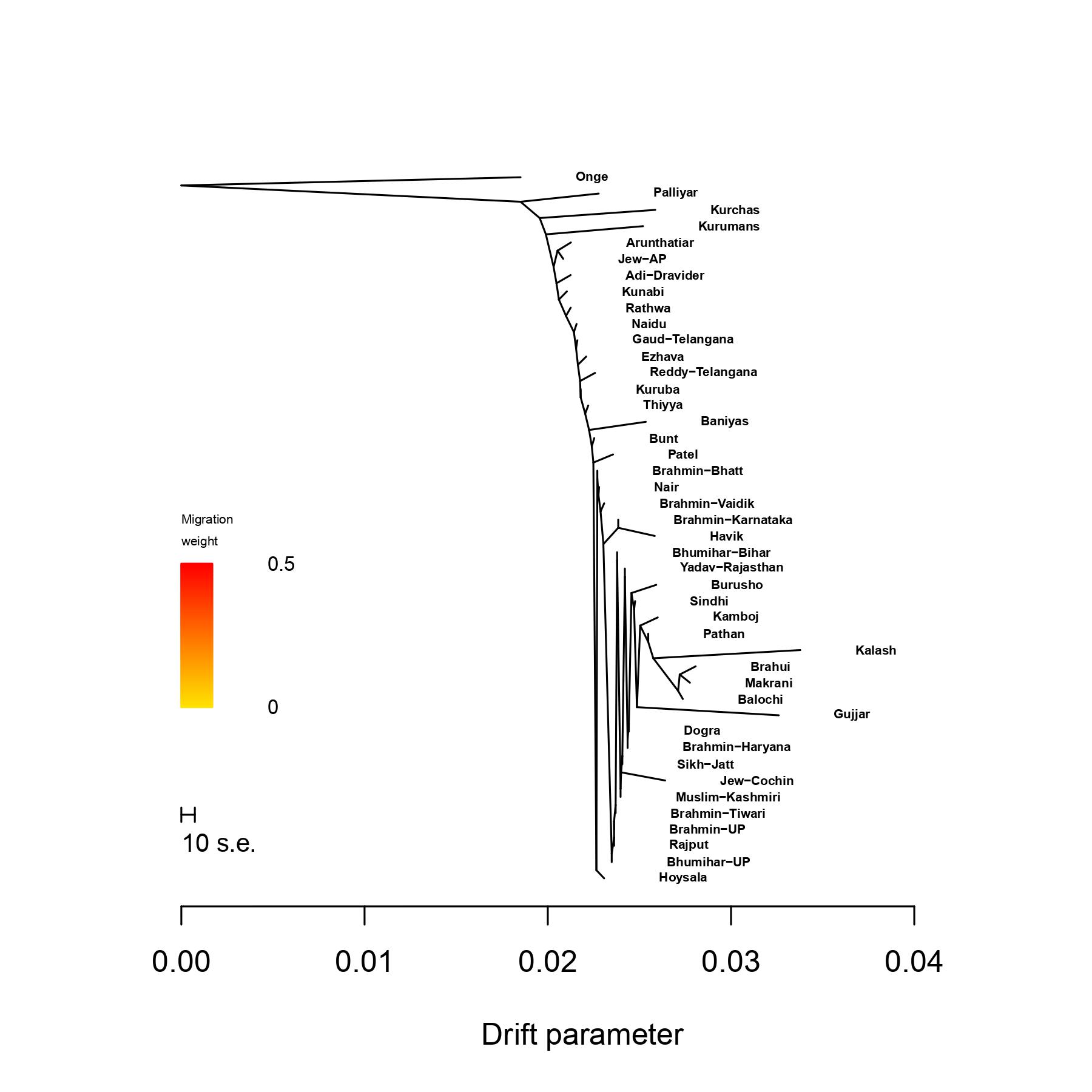


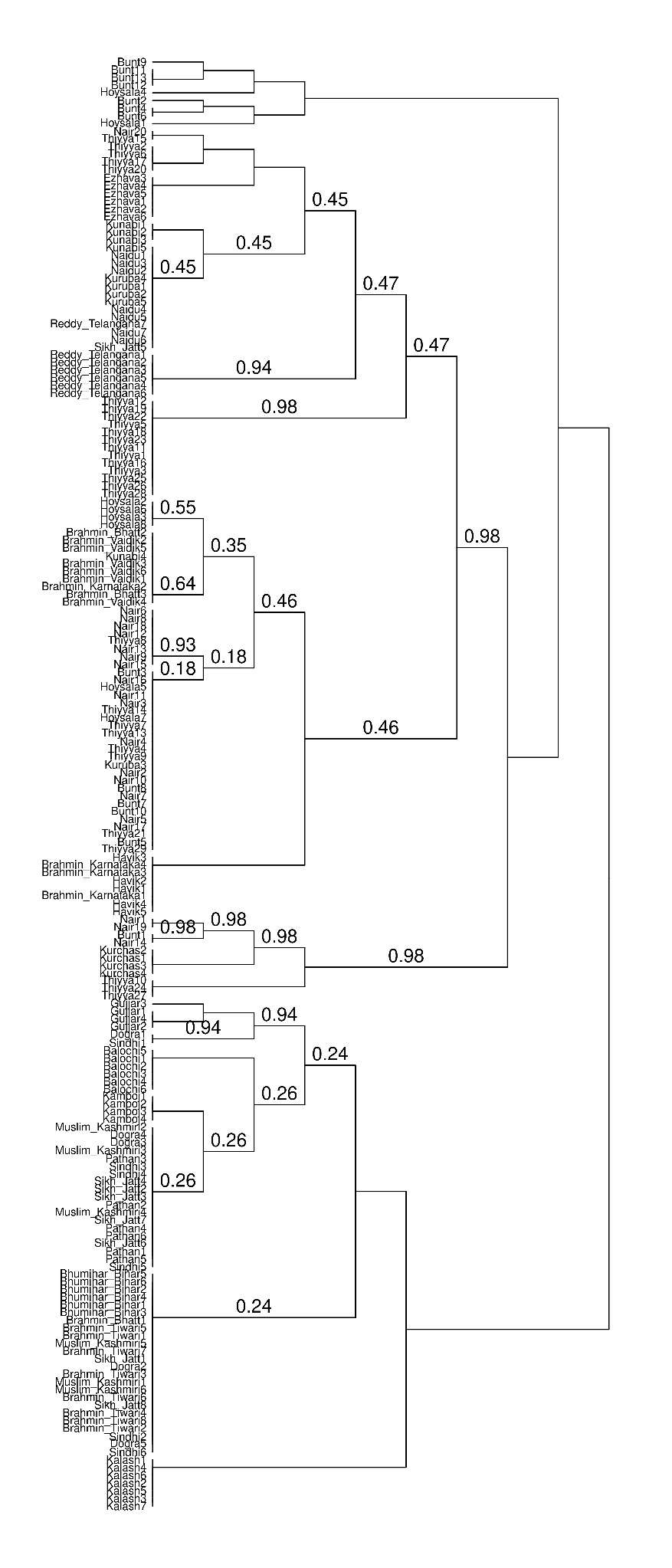
**Fig S4a.** Individual level phylogenetic tree for south west coastal groups along with other Indian and Pakistani groups based on haplotype sharing and constructed by FineStructure


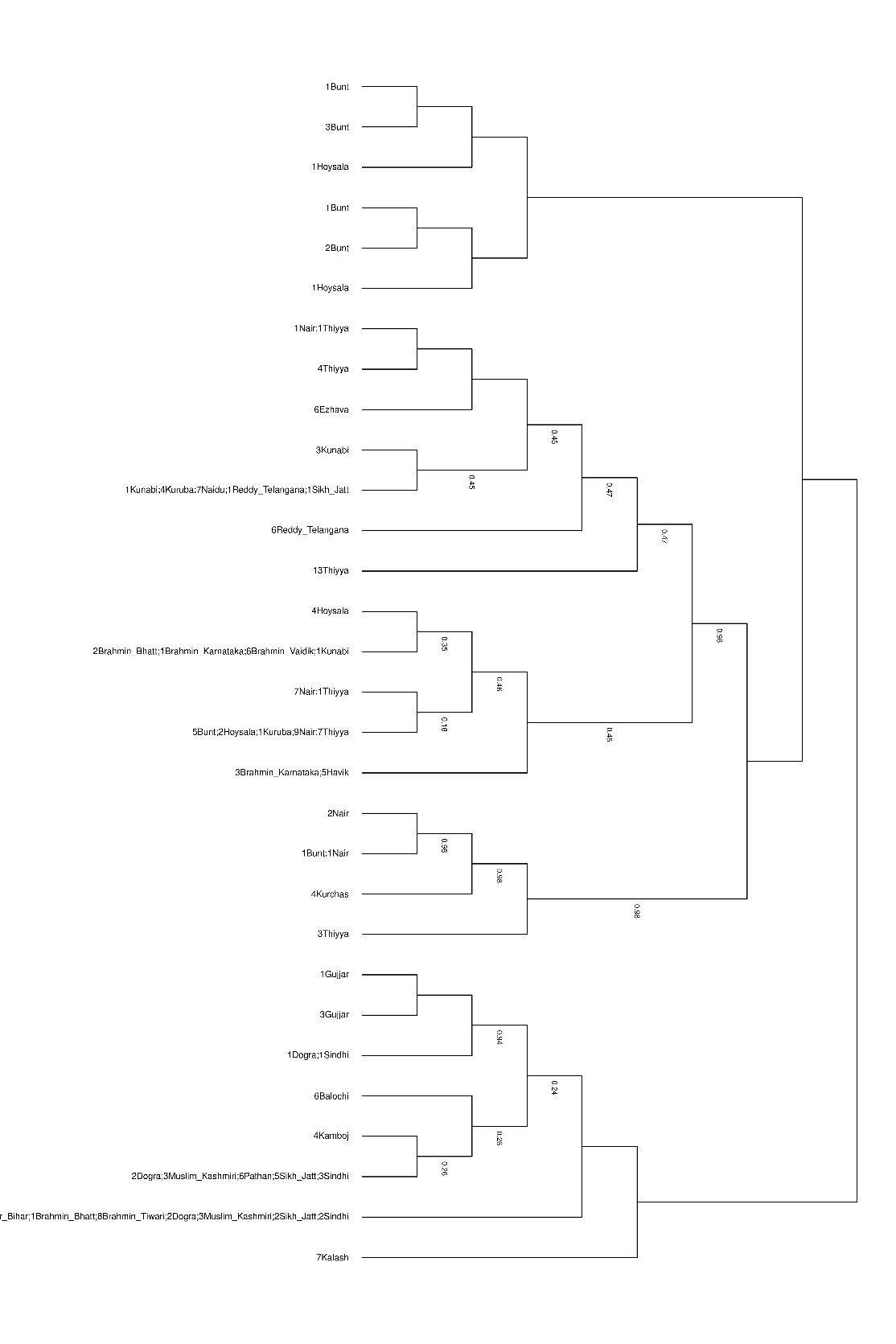
**Fig S4b.** Population level phylogenetic tree for south west coastal groups along with other Indian and Pakistani groups based on haplotype sharing and constructed by fineStructure

**Fig S5. Fitted admixture graph topology using qpGraph function in Admixtools 2.**

**A.** Fitted graph topology for Nair with ancient and some modern source groups showing additional Iranian component required from a distant ancestral source group related to BMAC. **B.** Fitted graph topology for Thiyya with ancient and some modern source groups. **C.** Fitted graph topology for Bunt with ancient and some modern source groups. **D.** Fitted graph topology for Ezhava with ancient and some modern source groups. **E.** Fitted graph topology for Gujjar with ancient and some modern source groups.


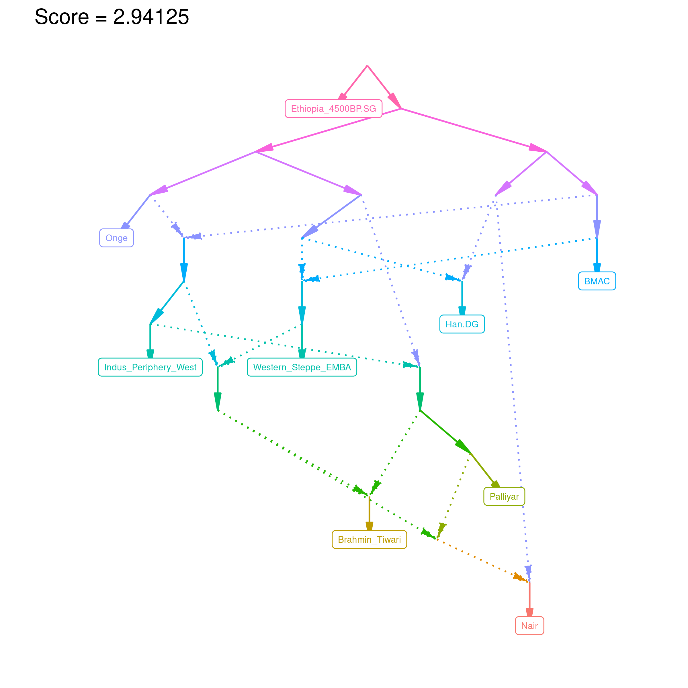


A


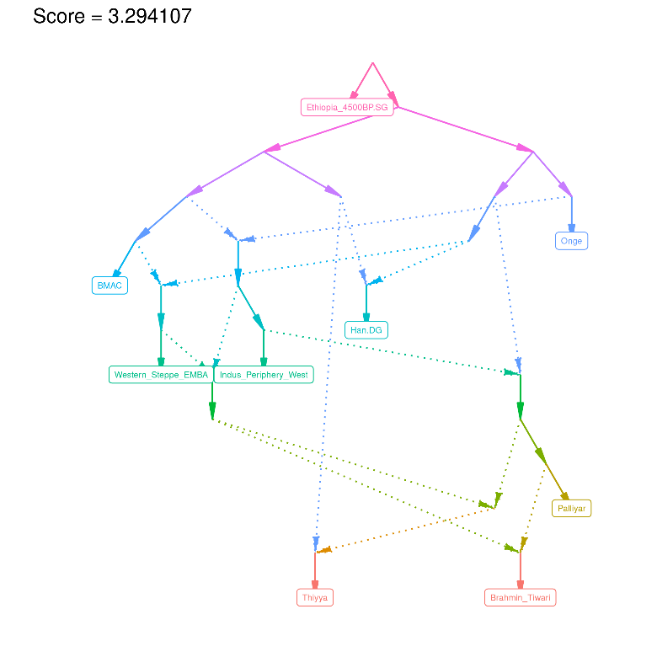


B


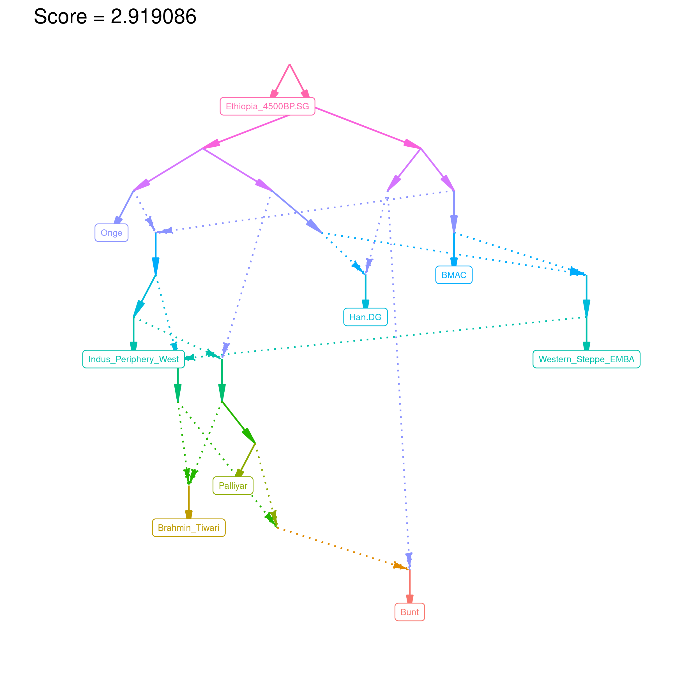


C


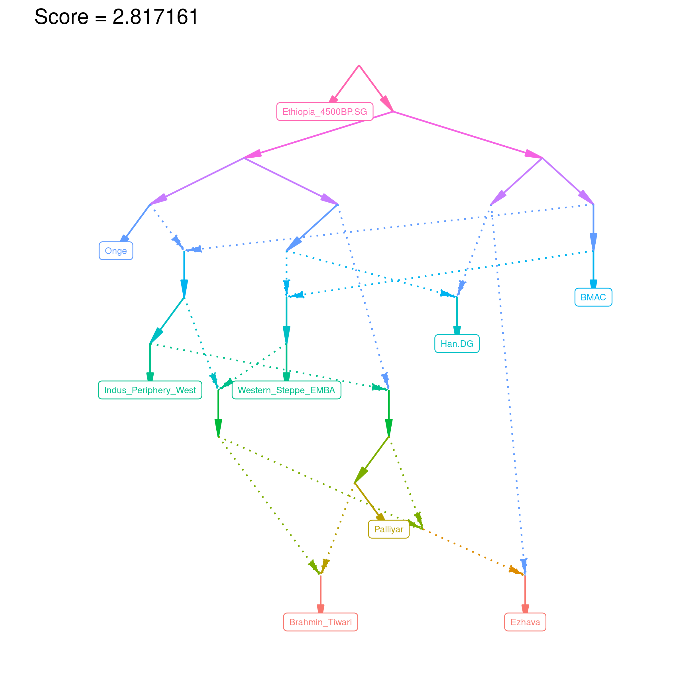


D


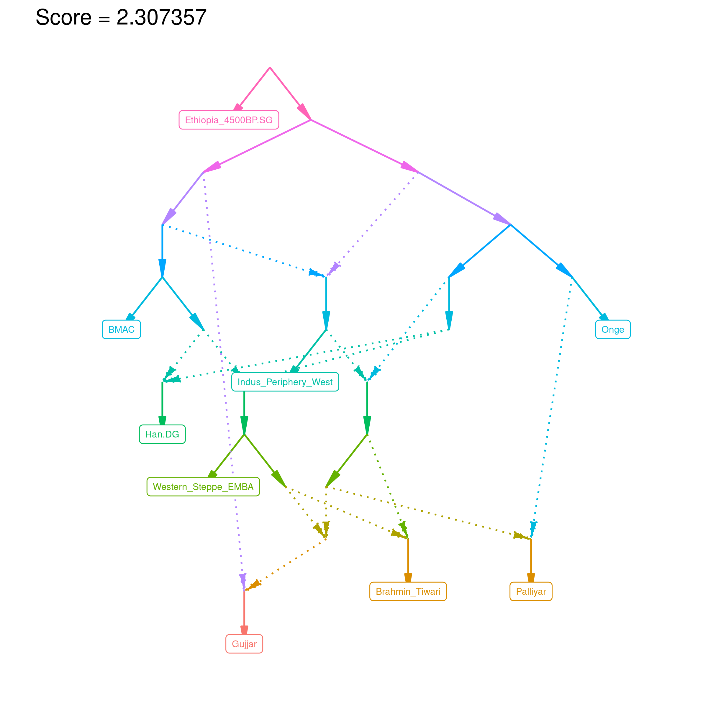


E

**Fig S6. Geographical population structure inferred from EEMS in the form of effective migration rates. A.** Contour plot for estimates of effective migration rates across the demes. **B.** A plot showing convergence of MCMC chains (1-10) used to infer posterior distribution of parameters. **C.** Plot showing a correlation between observed vs. fitted dissimilarity between demes.


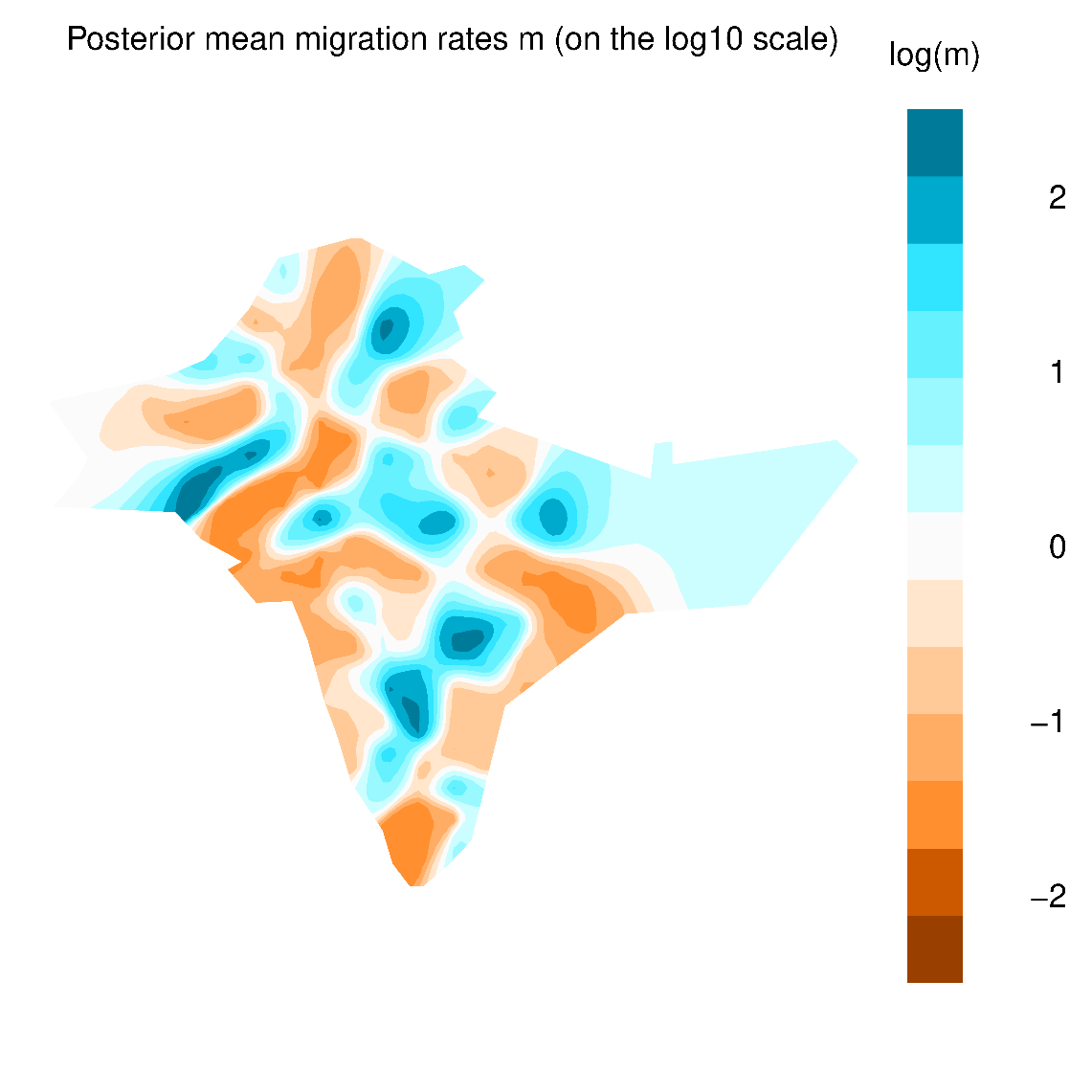


A

B

C


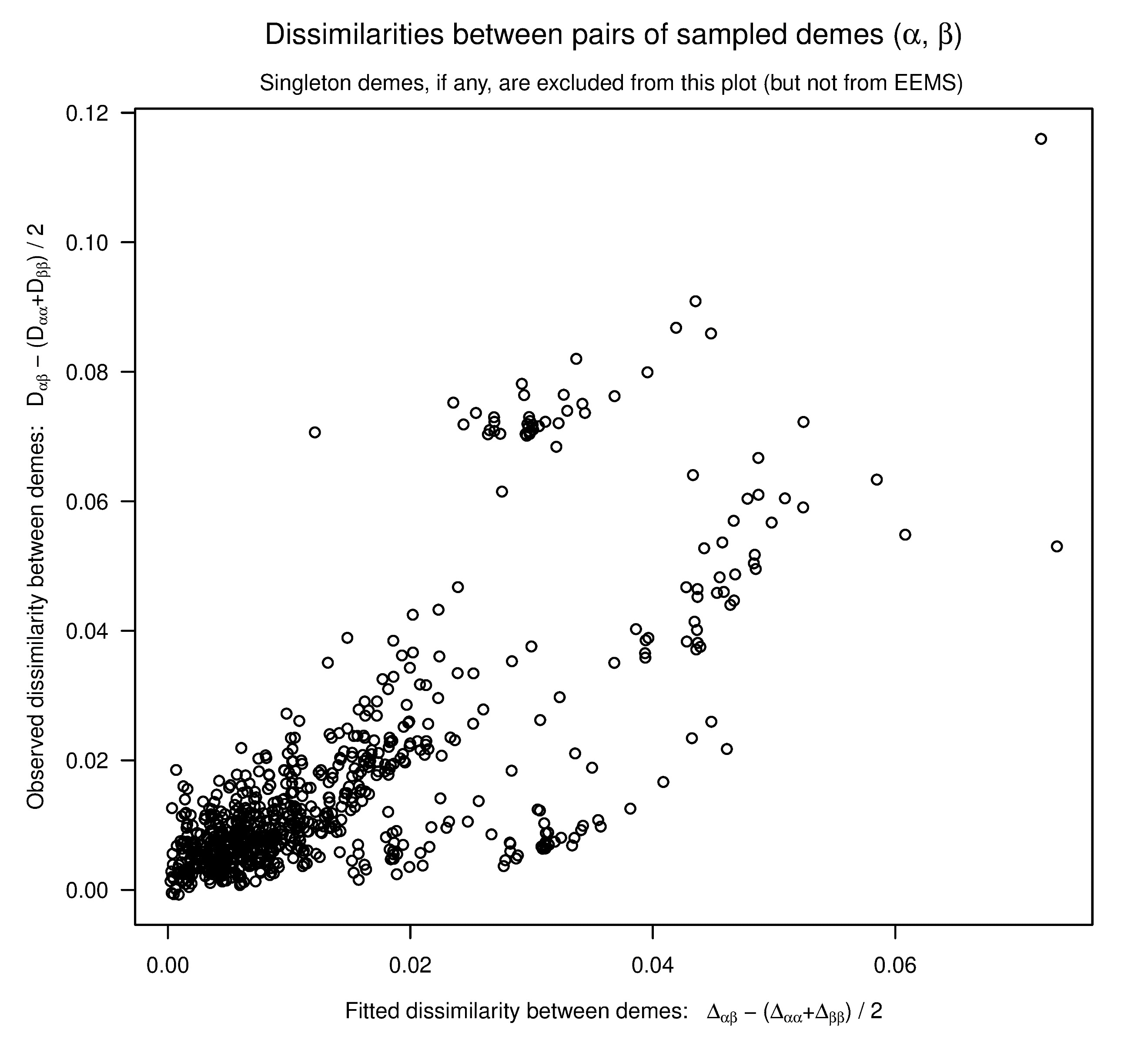

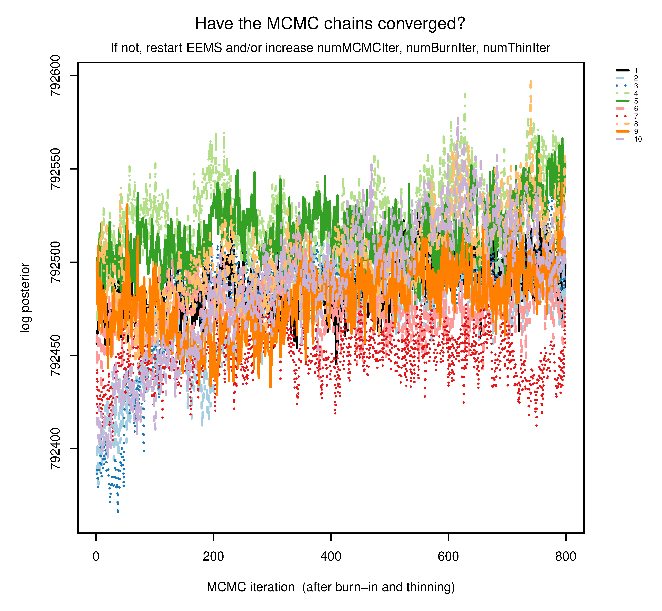


Fig S7. (A)Venn diagram of putative candidates of positive natural selection in all three selection scans with population pairs South West coast vs French; (B), (C) and (D) Manhattan plot for chr 2, Chr16 and chr 20 respectively.

A

D

C

B

**Fig S8.** **Mitochondrial haplogroup distribution among three major southwest coastal groups. A.** Distribution of macrohaplogroup among south-west coastal populations. **B.** Distribution of sub haplogroups of mitochondrial haplogroup M. **C.** Distribution of sub haplogroups of mitochondrial haplogroup U. **D.** Distribution of sub haplogroups of mitochondrial haplogroup R.


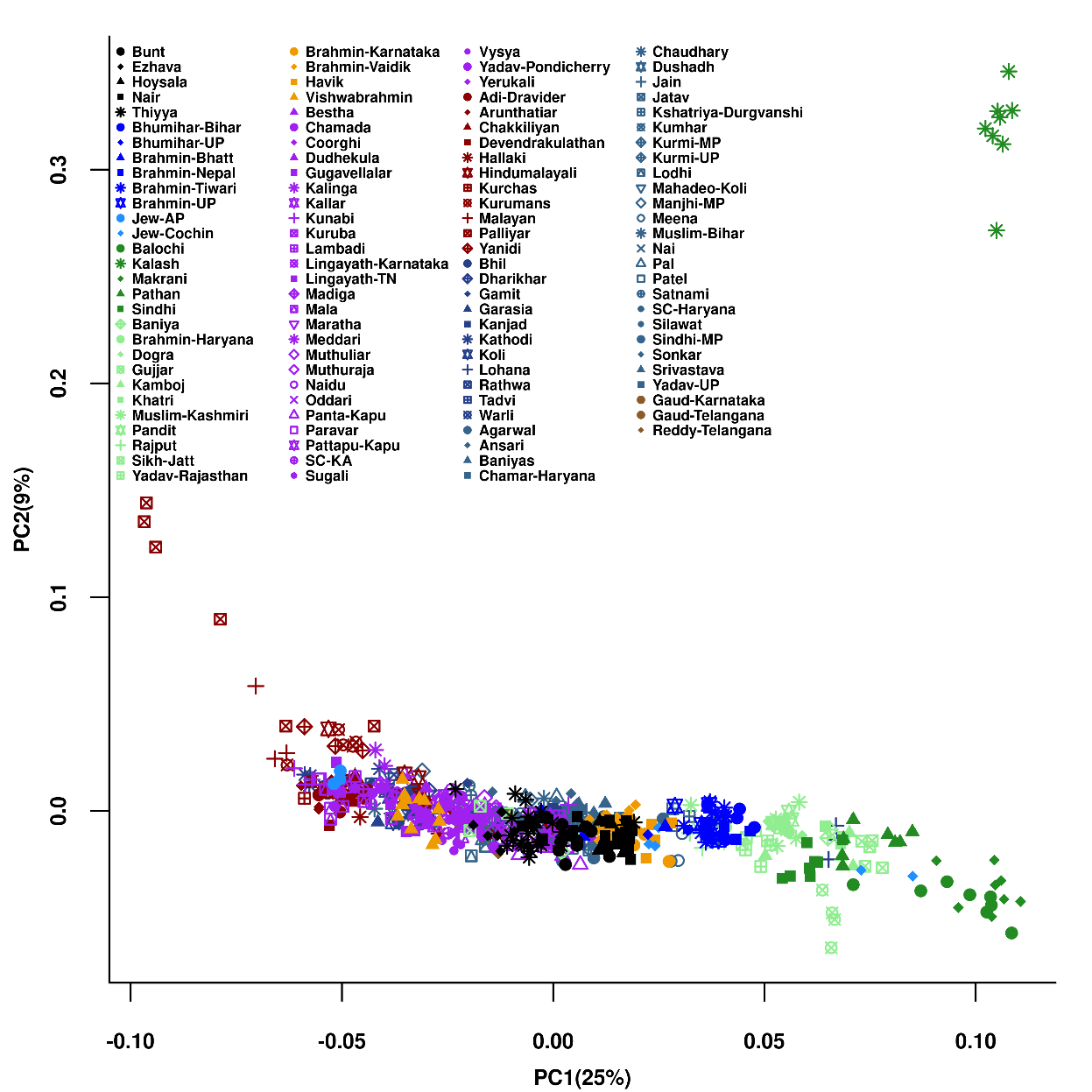
**Fig S9. PCA with South Asian groups on main ANI-ASI cline along with South West coast populations.**

References

Aiyappan A. 1944. Iravas and Culture Change: Superintendent, Government Press.

Fawcett F. 1915. Nayars of Malabar: Superintendent, Government Press.

Holland P, Club W. 1848. Pliny's Natural History. In Thirty-seven Books: Club.

Iyer LKAK. 1969. The Cochin Tribes and Castes: Johnson Reprint Corporation.

Jacob G. 1982. The History of the Awakening of the Ilava Community of Kerala. [

Menon AS. 2006. A Survey of Kerala History: S. Viswanathan.

Oldham CF. 1905. The Sun and the Serpent: A Contribution to the History of Serpent-worship: A. Constable & Company, Limited.

Sturrock J. 1894. Madras District Manuals: South Canara: Superintendent, Government Press.

Thurston E, Rangachari K. 2018. Castes and Tribes of Southern India; Volume 7: Creative Media Partners, LLC.
